# Supplementary material for: Epigenetic inheritance is unfaithful at intermediately methylated CpG sites
Source: Nat Commun. 2023 Sep 2;14:5336. doi: 10.1038/s41467-023-40845-2 (PMC10475082; doi:10.1038/s41467-023-40845-2)
Supplement: Supplementary file 1 — Supplementary Information [file 41467_2023_40845_MOESM1_ESM.pdf]

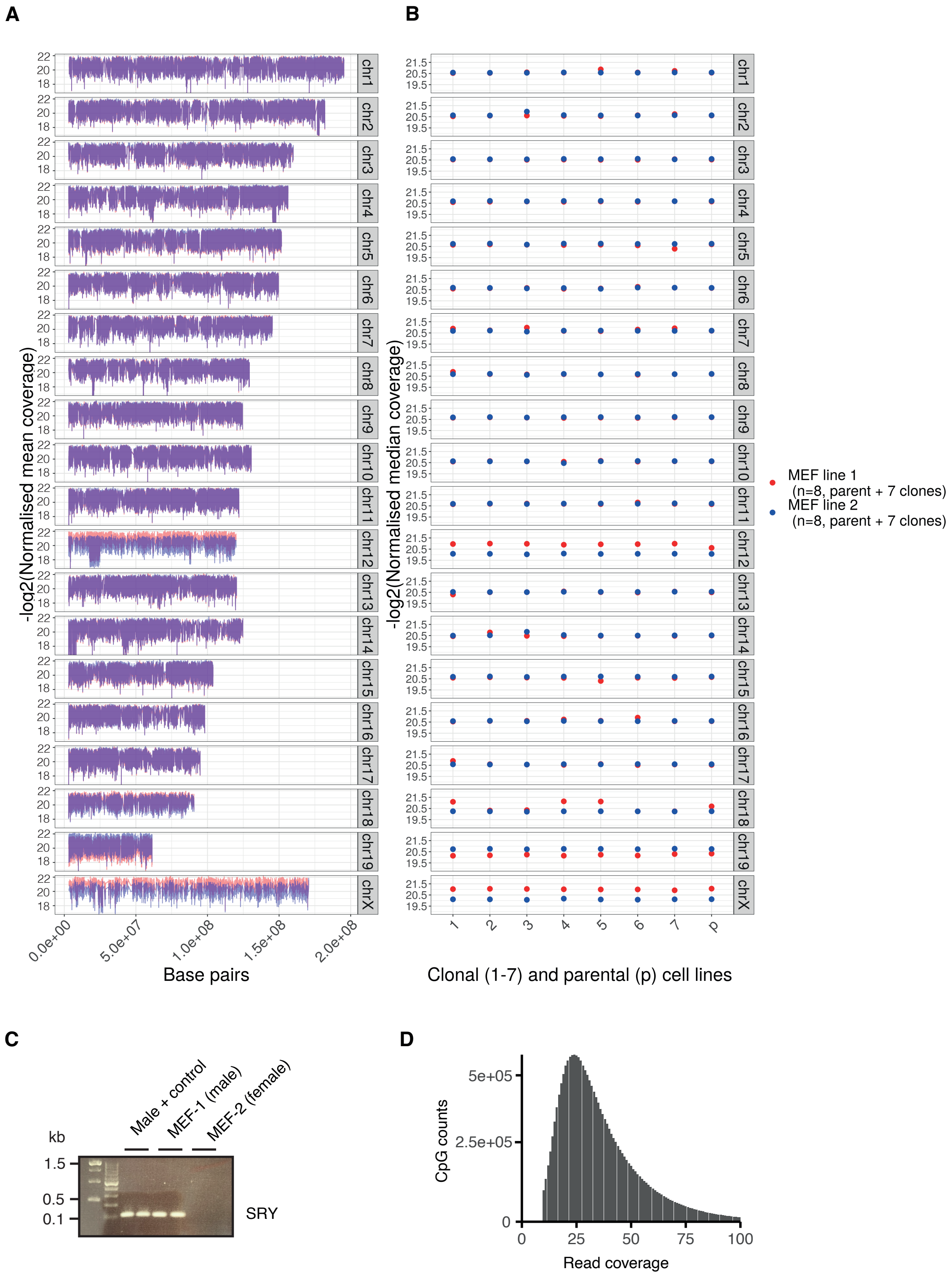

Supplementary Fig. 1: Filtering and thresholding MEF methylation data. A) Mean coverage (log2 normalised) across MEF-1 and MEF-2 as well as (B) the median coverage (log2 normalised) for each of the individual libraries. Decreased coverage across chromosome 12 and 18 for all or some of the MEF-1 lines compared to the MEF-2 lines suggests monosomies for those two chromosomes in some or all the MEF-1 lines. Increased coverage across chromosome 19 for all the MEF-1 lines suggests trisomy 19 for all the MEF-2 lines. For subsequent analyses, the CpGs on chromosomes 12, 18, and 19 were filtered out. Increased coverage on the X chromosome for all the MEF-2 lines suggests that these lines were derived from a female embryo. (C) Sex of the two lines was independently determined by PCR of the SRY gene (with two biological replicates), which confirmed that MEF-1 was derived from a male embryo and MEF-2 from a female embryo. For subsequent analyses, the CpGs on chromosomes X and Y were filtered out. (D) Thresholding data at greater than 10x coverage in all 16 methylation datasets results in a median coverage of 32 reads per CpG.

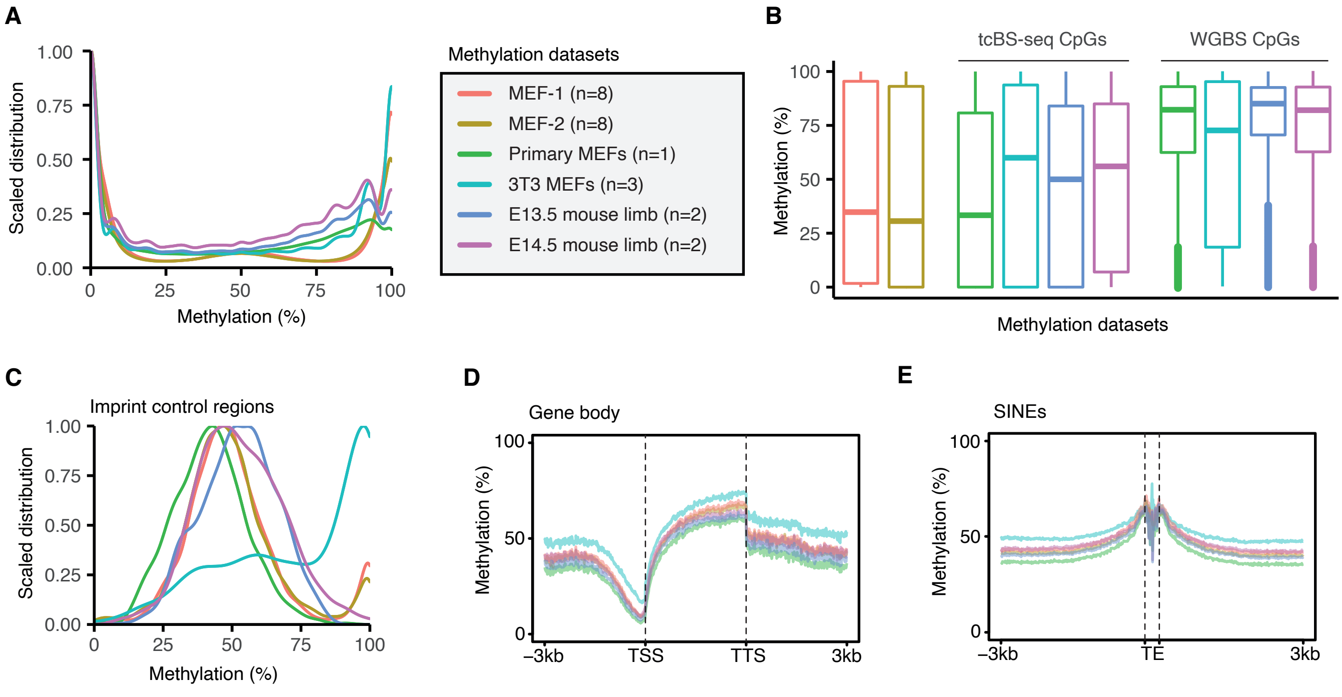

Supplementary Fig. 2: Validation of MEF target capture bisulfite sequencing. A) Density plots to show methylation distribution of the thresholded and filtered tc-BSseq CpGs for MEF-1, MEF-2, and relevant publicly available whole genome bisulfite sequencing (WGBS) datasets (see Supplementary Table 3). All datasets have comparable profiles of methylation for the tcBS-seq CpGs. The accompanying legend applies to all panels of this figure. (B) Boxplots of methylation distribution for MEF-1 and MEF-2 datasets (n = 1,203,687) compared to publicly available whole genome bisulfite sequencing (WGBS; n = 21,353,092) datasets that are also filtered for the tcBS-seq CpGs (n = 1,203,687). MEF-1 and MEF-2 have a similar distribution to the primary MEFs when filtered for the tcBS-seq CpGs, but a lower distribution when considering CpGs genome-wide. (C) Methylation distributions of CpGs at imprint control regions are similar across all datasets, aside from the 3T3 immortalised MEFs. (D) Methylation shows a similar pattern of reduced values at TSSs and enrichment across gene bodies in all datasets when filtered for the tcBS-seq CpGs. (E) Methylation is enriched at the 5' and 3' edges of SINEs in all datasets when filtered for the tcBS-seq CpGs. For (B), the box of the boxplot shows the 25th, 50th, and 75th percentiles; the whiskers extend to 1.5 * IQR beyond the edges of the box (where IQR = 75th–25th percentile), with outliers shown as dots.(D) and (E) were generated using SeqPlots (v3.0.12).

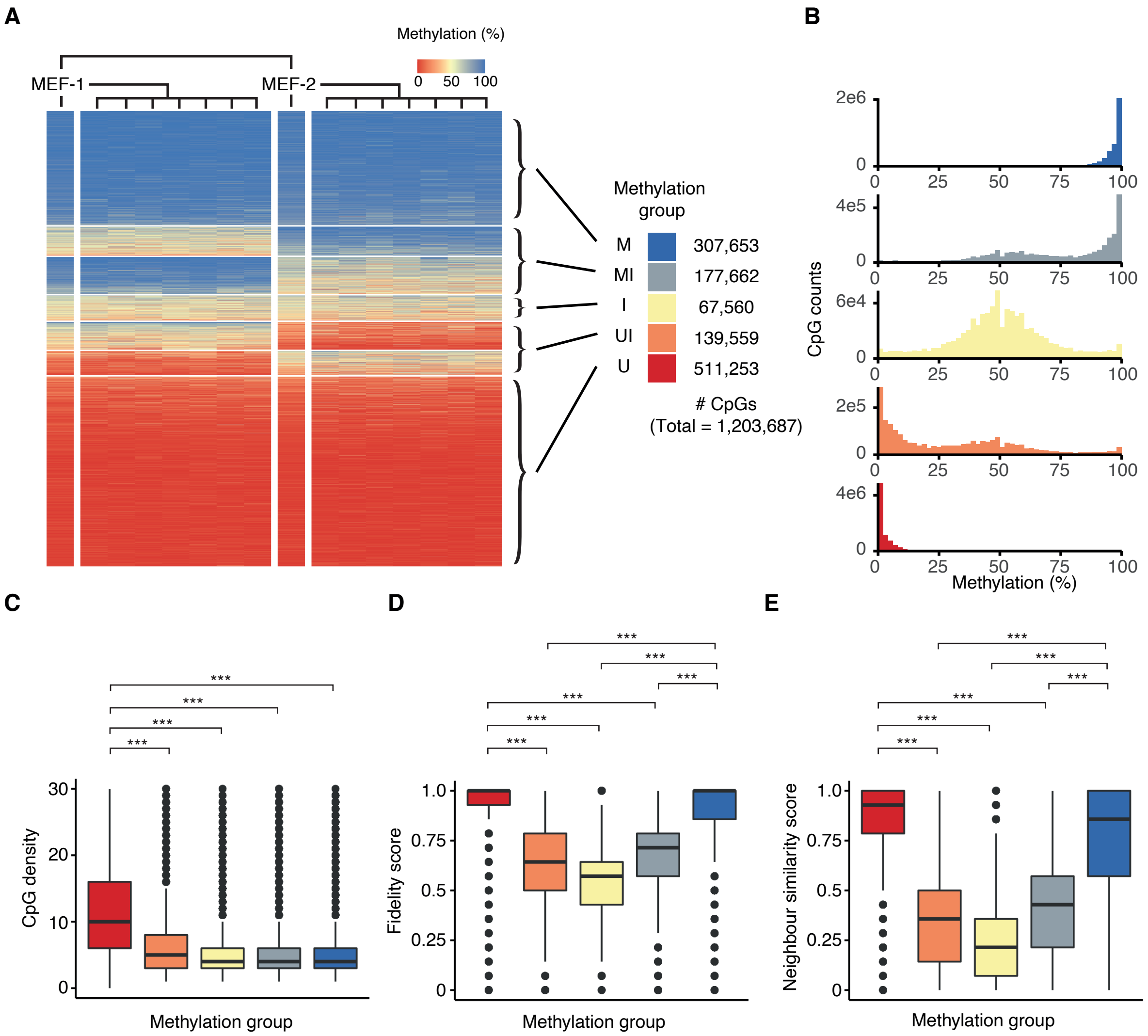

Supplementary Fig. 3: Classifying and evaluating methylation groups in MEFs. A) Classifying five methylation groups for 1,203,687 CpGs from the seven k-means clusters of MEF-1 and MEF-2 parental and clonal line methylation data (as shown in Fig. 1C). U = consistently hypomethylated across all the cell lines (n = 511,253). UI = potential to be either hypo- or intermediately methylated (n = 139,559). I = intermediately methylated (n = 67,560). MI = potential to be either hyper- or intermediately methylated (n = 177,662). M = consistently hypermethylated across all the cell lines (n = 307,653). (B) Methylation distributions of the methylation groups across all MEF datasets. (C) Comparing CpG density per 100bp, (D) fidelity score, and (E) neighbour similarity score of the different methylation groups. ***p < 2e-16. P-values for (C) are from quasi-Poisson regressions (one-sided); p-values for (D) and (E) are from Wilcoxon rank-sum tests (two-sided). For (C), (D), and (E), the box of the boxplot shows the 25th, 50th, and 75th percentiles; the whiskers extend to 1.5 * IQR beyond the edges of the box (where IQR = 75th–25th percentile), with outliers shown as dots.

Supplementary Figures 4-19

Examples of unfaithful, faithful, and imprinted regions of the genome. The parental lines (either MEF-1 or MEF-2) are shown to the left, with the seven clonal lines shown to the right. Reads from the tcBS-seq data are represented by lines and CpGs are shown as circles with accurate mapping of respective distance in base pairs between them. Methylated CpGs are shown as filled circles, while unmethylated CpGs are shown as open circles. Methylation percentages for each CpG are shown above the reads. CpG sites having fewer than 10 methylation calls across the samples in each cell line were excluded, and reads having fewer than 3 covered CpGs were excluded. Reads were then ordered by average methylation of covered CpG sites. Unfaithful regions (≥ 5 bp) were identified with an average fidelity score < 0.75 across at least 3 CpGs. Faithful regions (≥ 5 bp) were identified with an average fidelity score = 1 across at least 3 CpGs.

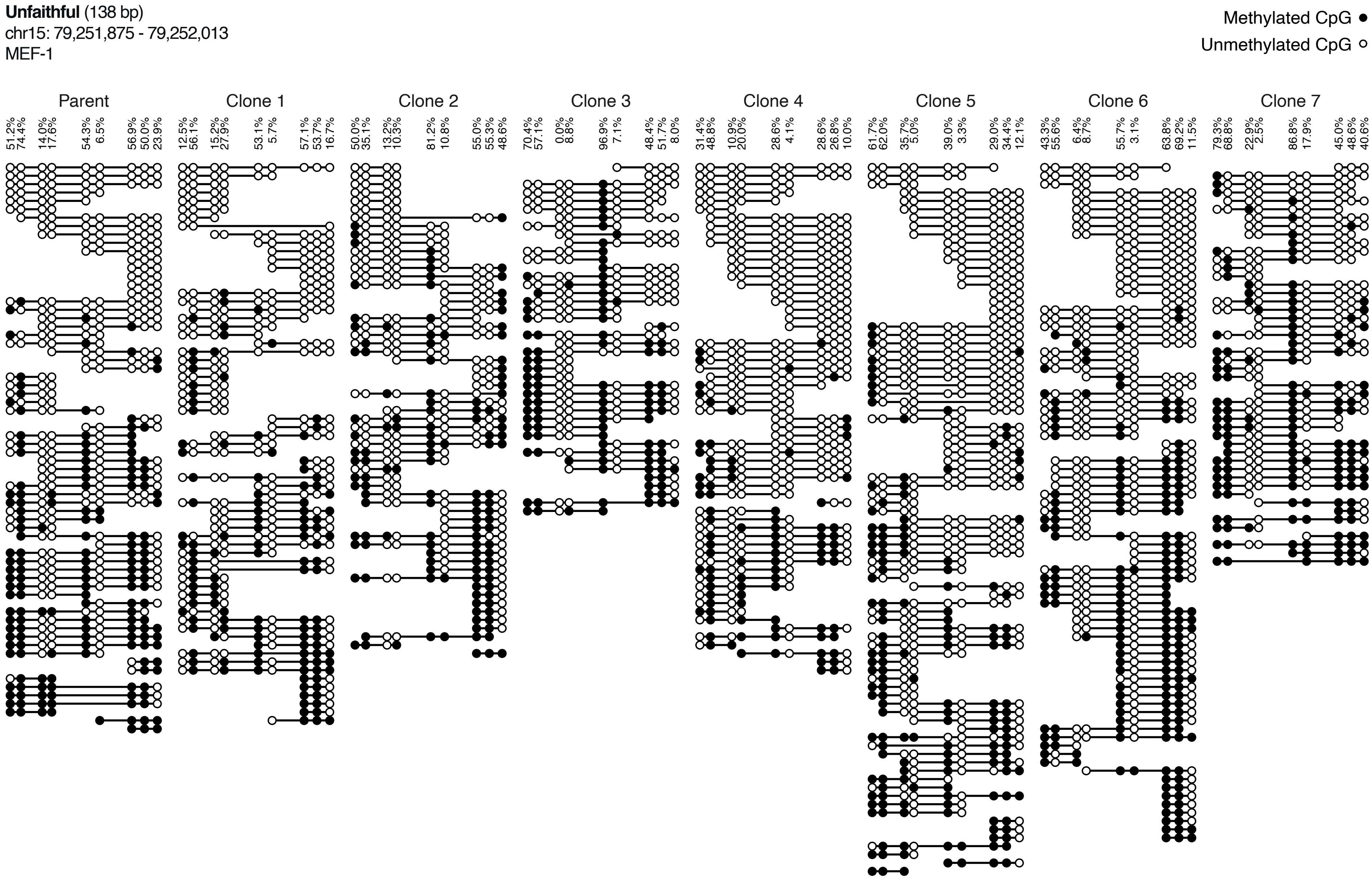

Unfaithful (138 bp)
chr15: 79,251,875 - 79,252,013
MEF-1
Methylated CpG
Unmethylated CpG
Parent
51.2% 74.4% 14.0% 17.6% 54.3% 6.5% 56.9% 50.0% 23.9%
Clone 1
12.5% 56.1% 15.2% 27.9% 53.1% 5.7% 57.1% 53.7% 16.7%
Clone 2
50.0% 35.1% 13.2% 10.3% 81.2% 10.8% 55.0% 55.3% 48.6%
Clone 3
70.4% 57.1% 0.0% 8.8% 96.9% 7.1% 48.4% 51.7% 8.0%
Clone 4
31.4% 48.8% 10.9% 20.0% 28.6% 4.1% 28.6% 26.8% 10.0%
Clone 5
61.7% 62.0% 35.7% 5.0% 39.0% 3.3% 29.0% 34.4% 12.1%
Clone 6
43.3% 55.6% 6.4% 8.7% 55.7% 3.1% 63.8% 69.2% 11.5%
Clone 7
79.3% 68.8% 22.9% 2.5% 86.8% 17.9% 45.0% 48.6% 40.6%

Unfaithful (63 bp)
chr7: 40,902,432 - 40,902,495
MEF-1

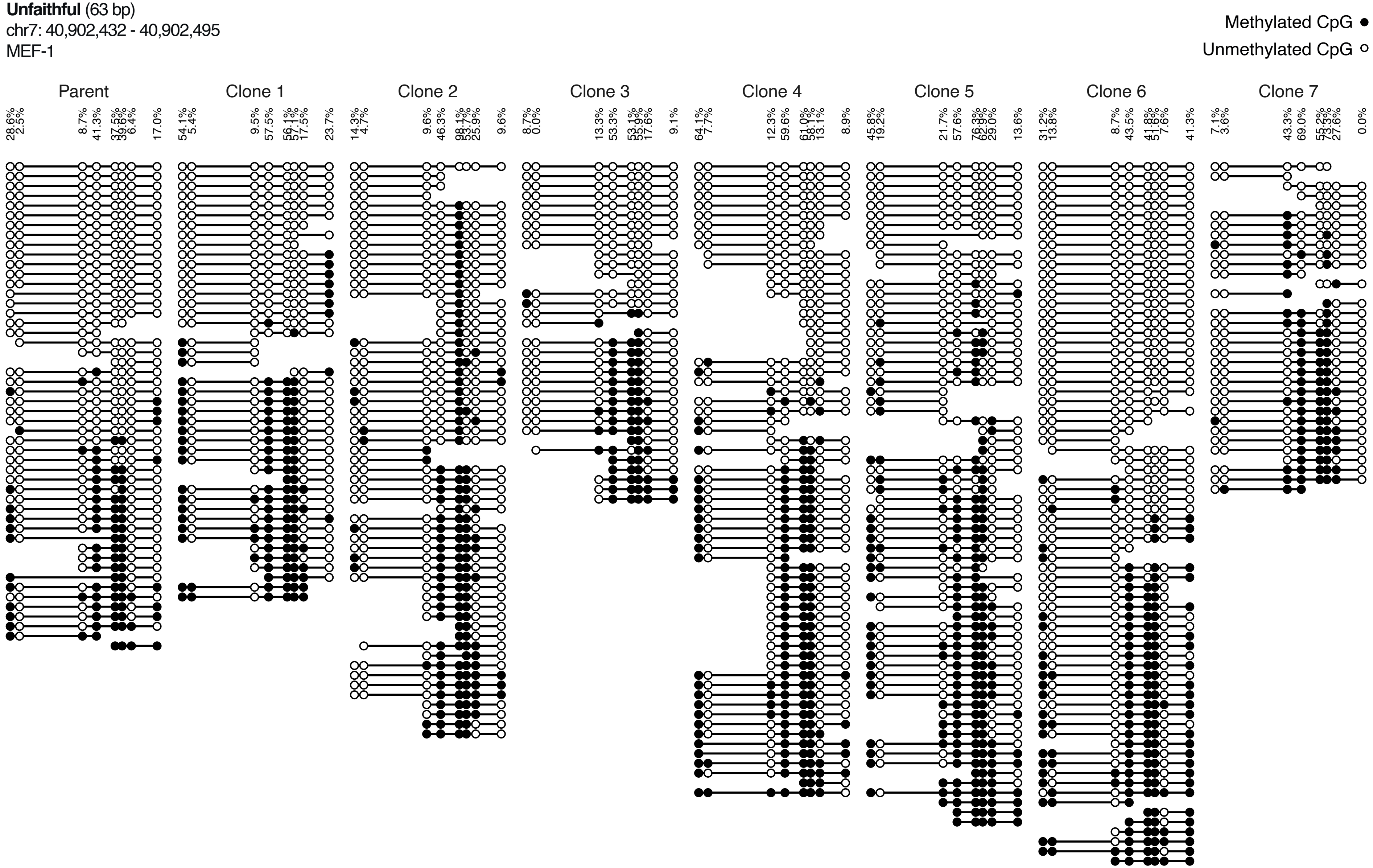

Unfaithful (138 bp)
chr15: 79,251,875 - 79,252,013
MEF-2

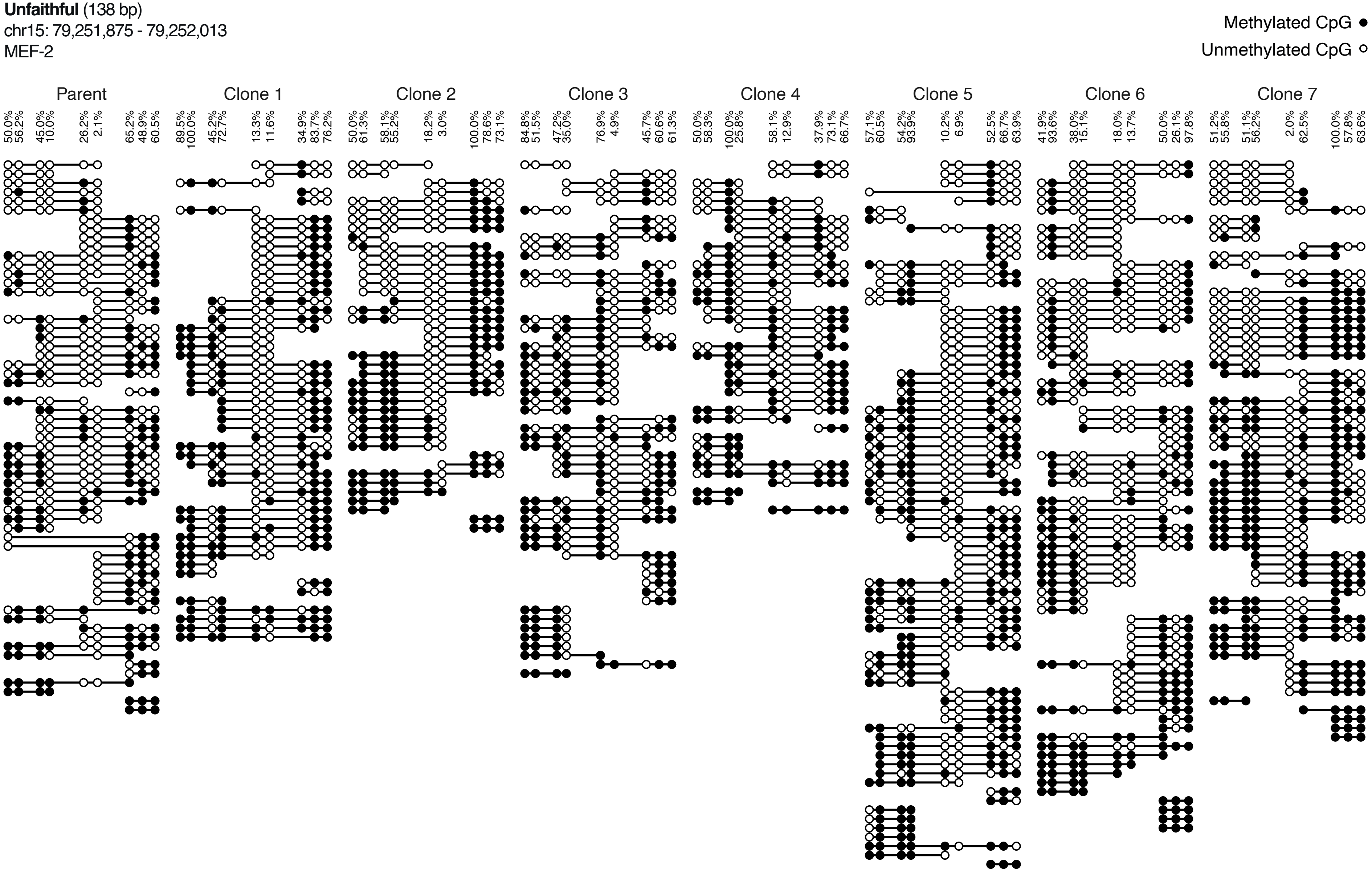

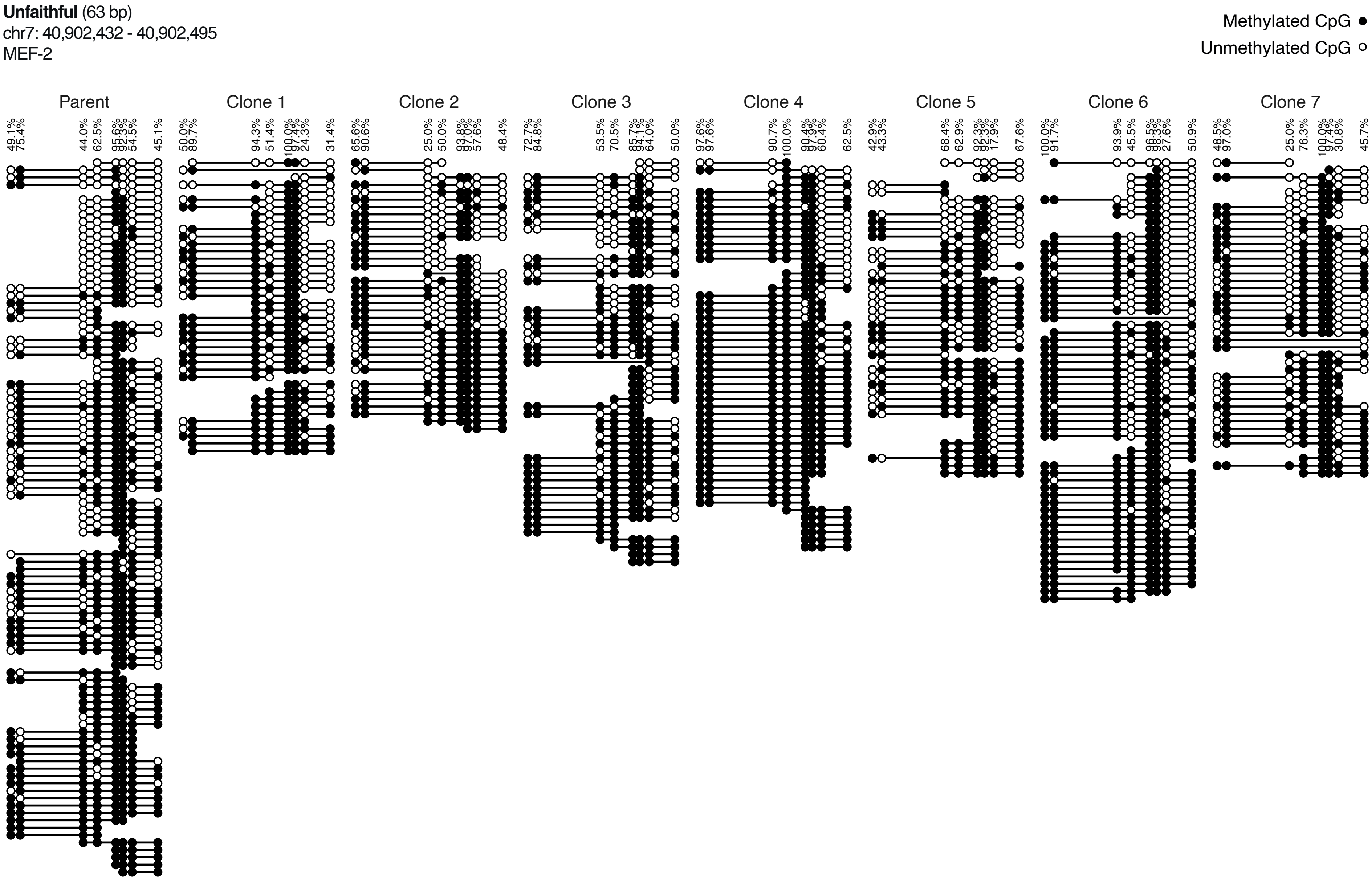
Unfaithful (63 bp)
chr7: 40,902,432 - 40,902,495
MEF-2
Methylated CpG ●
Unmethylated CpG ○
Parent
Clone 1
Clone 2
Clone 3
Clone 4
Clone 5
Clone 6
Clone 7

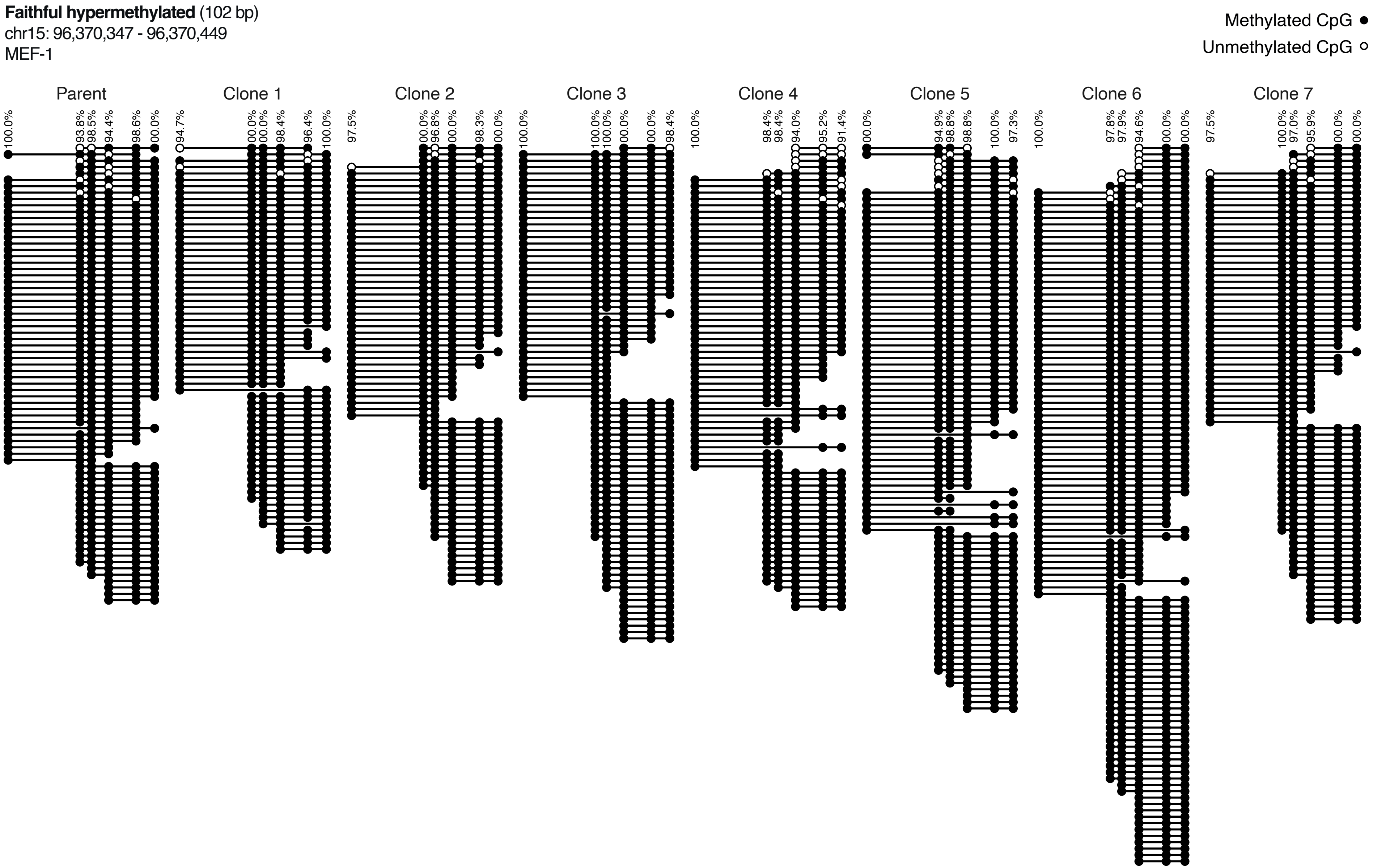
Faithful hypermethylated (102 bp)
chr15: 96,370,347 - 96,370,449
MEF-1
Methylated CpG
Unmethylated CpG
Parent
100.0% 93.8% 98.5% 94.4% 98.6% 00.0%
Clone 1
94.7% 00.0% 00.0% 98.4% 96.4% 100.0%
Clone 2
97.5% 00.0% 96.8% 00.0% 98.3% 00.0%
Clone 3
100.0% 100.0% 100.0% 00.0% 00.0% 98.4%
Clone 4
100.0% 98.4% 98.4% 94.0% 95.2% 91.4%
Clone 5
00.0% 94.9% 98.8% 98.8% 100.0% 97.3%
Clone 6
100.0% 97.8% 97.9% 94.6% 00.0% 00.0%
Clone 7
97.5% 100.0% 97.0% 95.9% 00.0% 00.0%

Faithful hypermethylated (17 bp)
chr1: 34,302,911 - 34,302,928
MEF-1

Methylated CpG ●
Unmethylated CpG ○

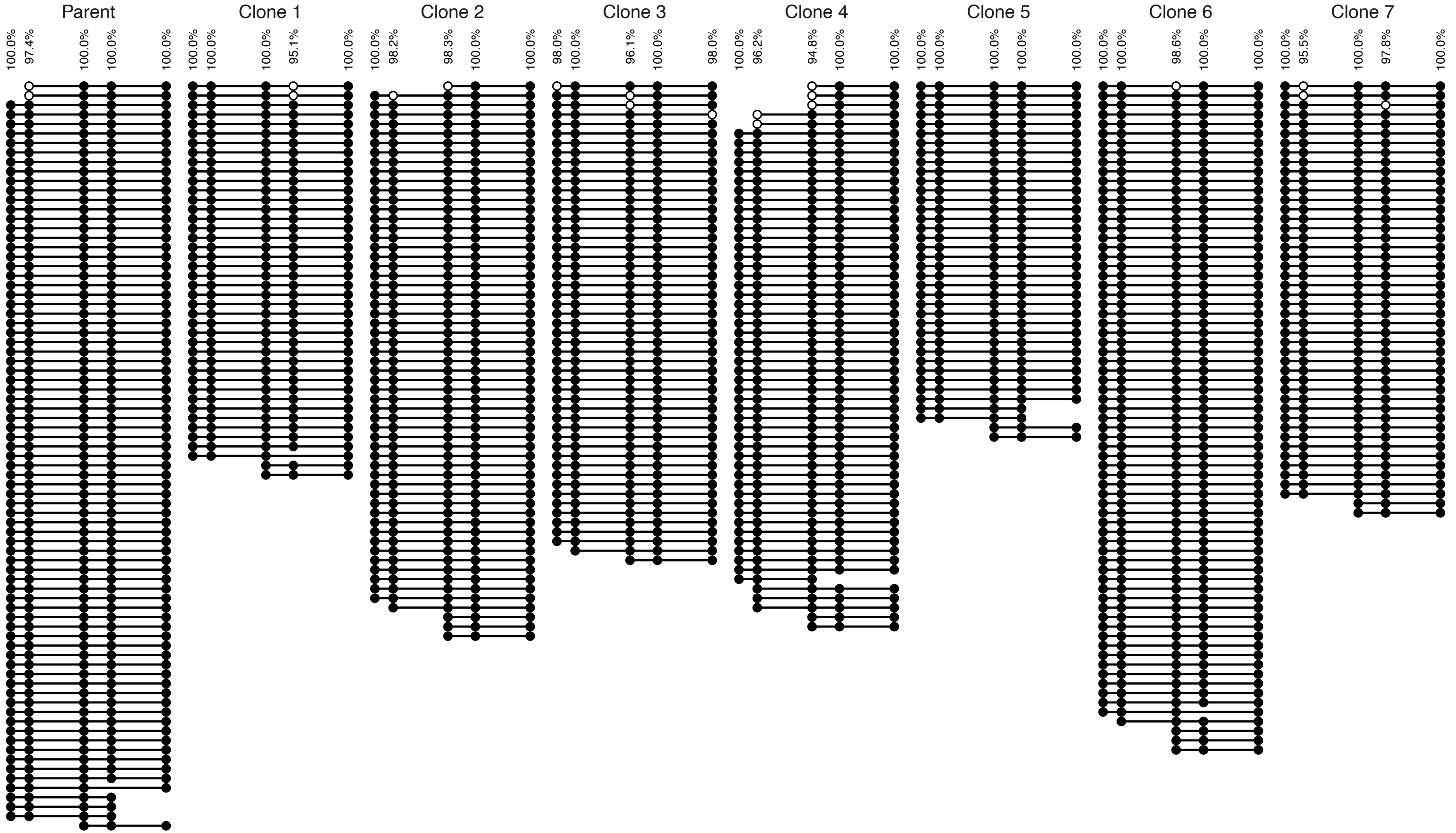

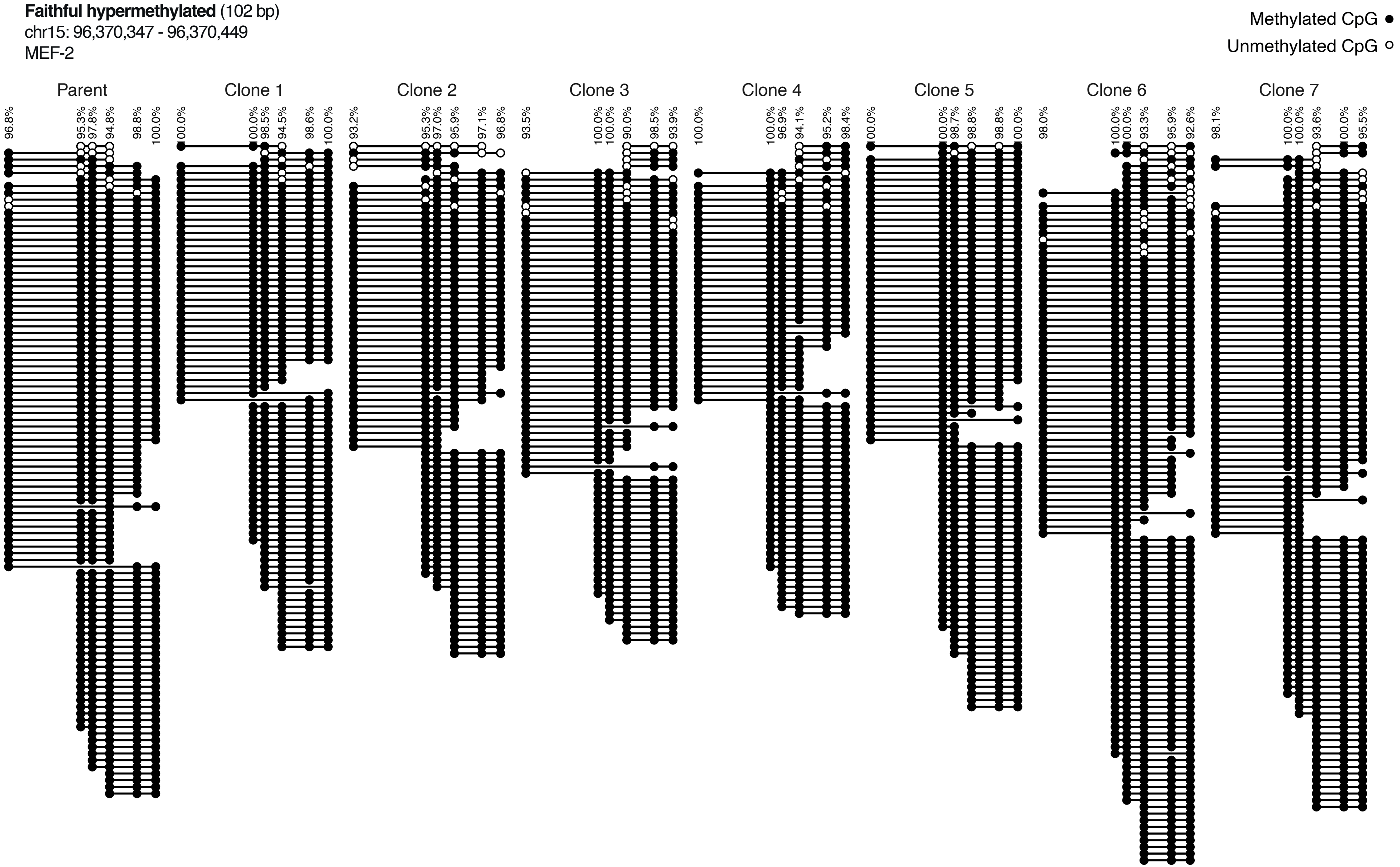
Faithful hypermethylated (102 bp)
chr15: 96,370,347 - 96,370,449
MEF-2
Methylated CpG
Unmethylated CpG
Parent
96.8% 95.3% 97.8% 94.8% 98.8% 100.0%
Clone 1
100.0% 100.0% 98.5% 94.5% 98.6% 100.0%
Clone 2
93.2% 95.3% 97.0% 95.9% 97.1% 96.8%
Clone 3
93.5% 100.0% 100.0% 90.0% 98.5% 93.9%
Clone 4
100.0% 100.0% 96.9% 94.1% 95.2% 98.4%
Clone 5
100.0% 100.0% 98.7% 98.8% 98.8% 100.0%
Clone 6
98.0% 100.0% 100.0% 93.3% 95.9% 92.6%
Clone 7
98.1% 100.0% 100.0% 93.6% 100.0% 95.5%

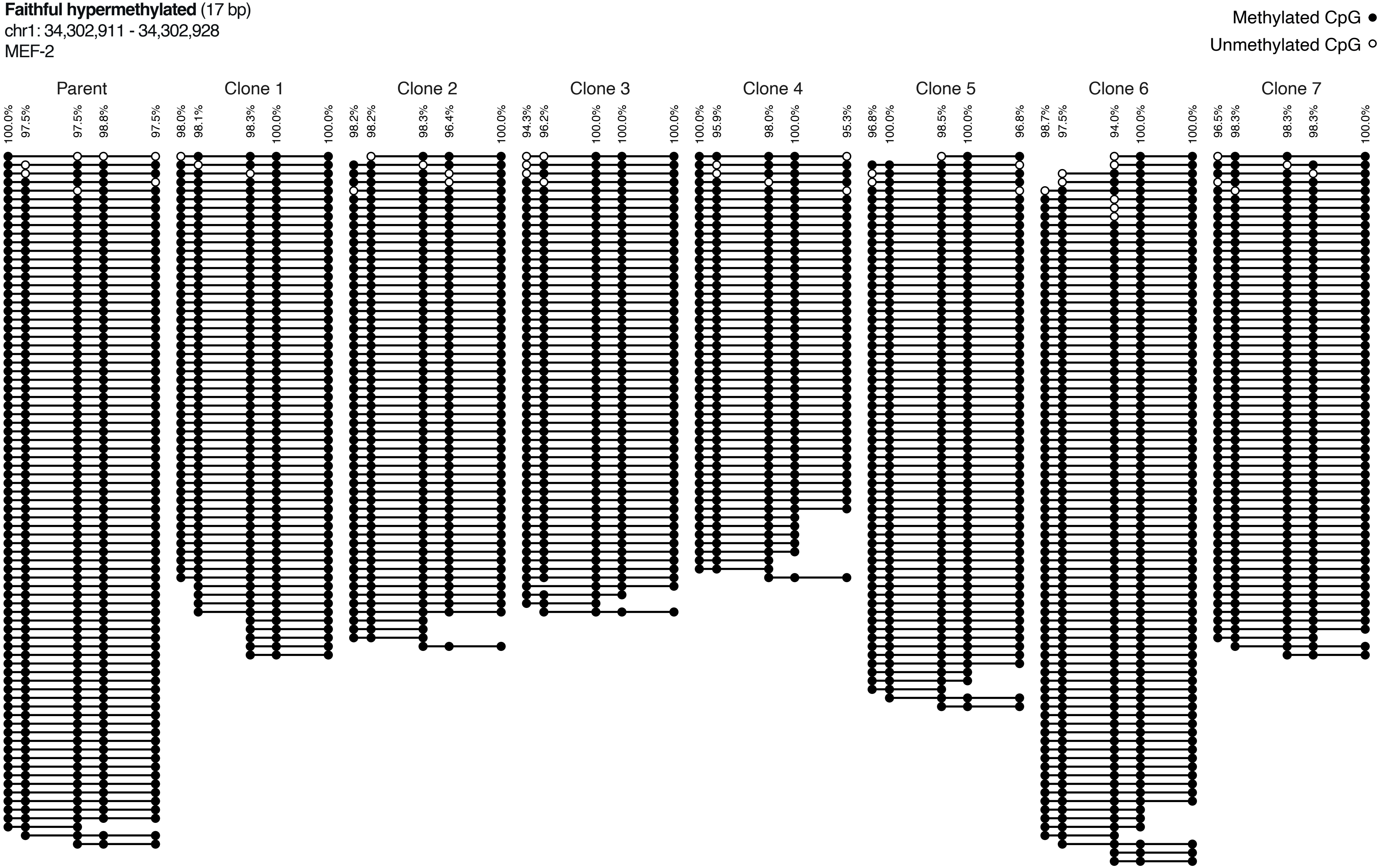
Faithful hypermethylated (17 bp)
chr1: 34,302,911 - 34,302,928
MEF-2
Methylated CpG
Unmethylated CpG
Parent
100.0% 97.5% 97.5% 98.8% 97.5%
Clone 1
98.0% 98.1% 98.3% 100.0% 100.0%
Clone 2
98.2% 98.2% 98.3% 96.4% 100.0%
Clone 3
94.3% 96.2% 100.0% 100.0% 100.0%
Clone 4
100.0% 95.9% 98.0% 100.0% 95.3%
Clone 5
96.8% 100.0% 98.5% 100.0% 96.8%
Clone 6
98.7% 97.5% 94.0% 100.0% 100.0%
Clone 7
96.5% 98.3% 98.3% 98.3% 100.0%

Faithful hypermethylated (29 bp)
chr4: 124,751,396 - 124,751,425
MEF-1

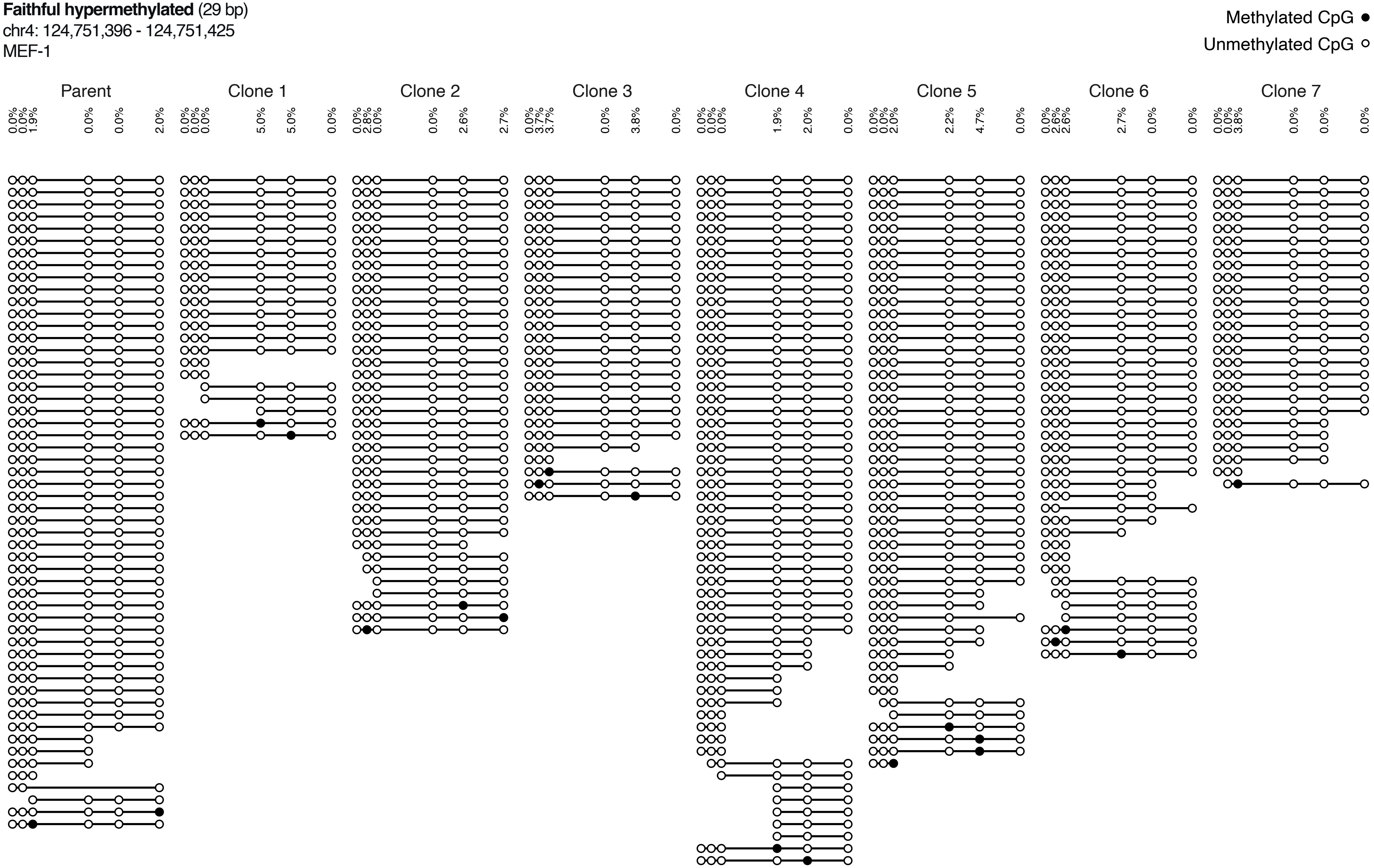

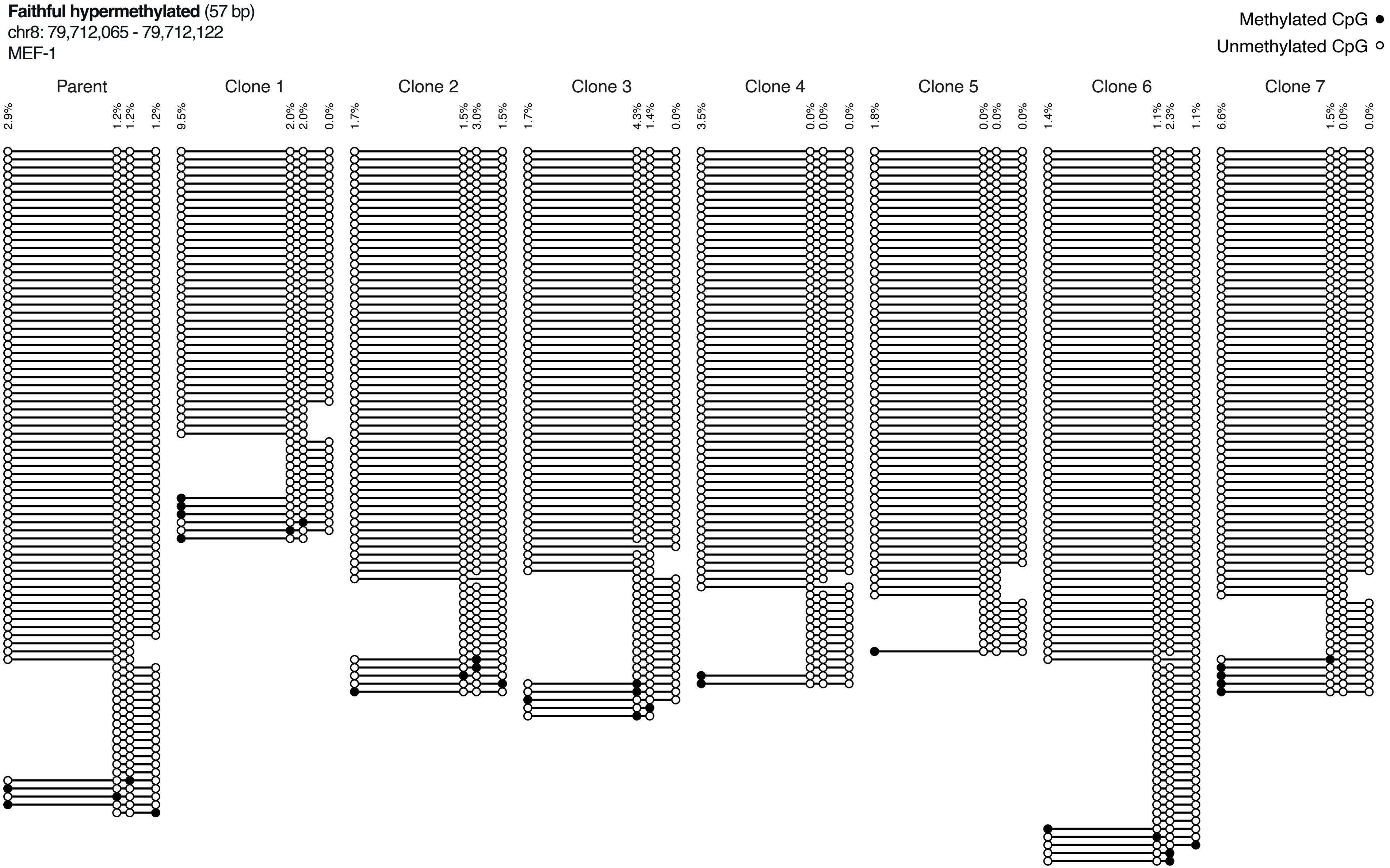
Faithful hypermethylated (57 bp)
chr8: 79,712,065 - 79,712,122
MEF-1
Methylated CpG
Unmethylated CpG
Parent
2.9%
1.2%
1.2%
1.2%
Clone 1
9.5%
2.0%
2.0%
0.0%
Clone 2
1.7%
1.5%
3.0%
1.5%
Clone 3
1.7%
4.3%
1.4%
0.0%
Clone 4
3.5%
0.0%
0.0%
0.0%
Clone 5
1.8%
0.0%
0.0%
0.0%
Clone 6
1.4%
1.1%
2.3%
1.1%
Clone 7
6.6%
1.5%
0.0%
0.0%

Faithful hypermethylated (29 bp)
chr4: 124,751,396 - 124,751,425
MEF-2

Methylated CpG ●
Unmethylated CpG ○

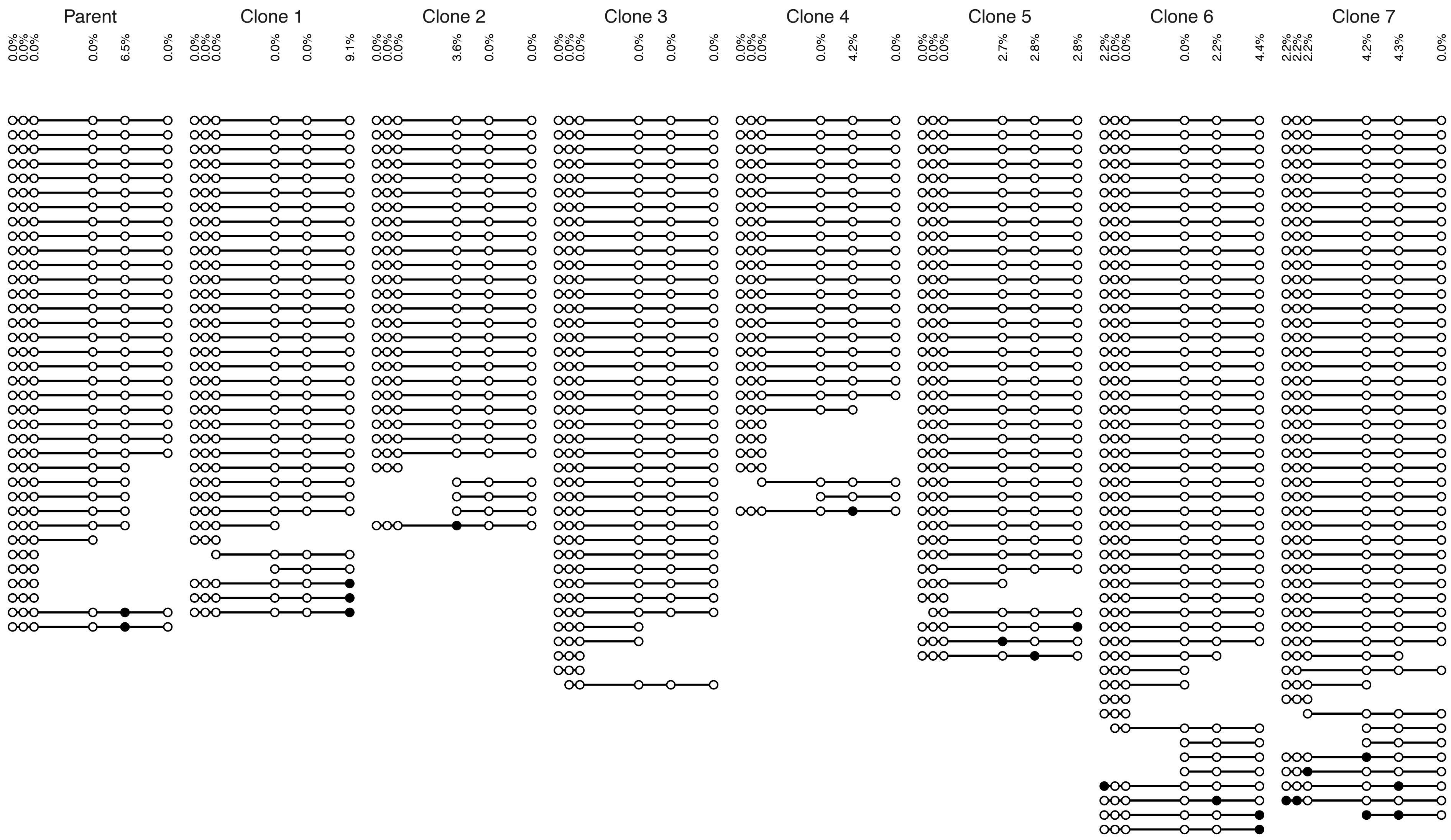

Faithful hypermethylated (57 bp)
chr8: 79,712,065 - 79,712,122
MEF-2

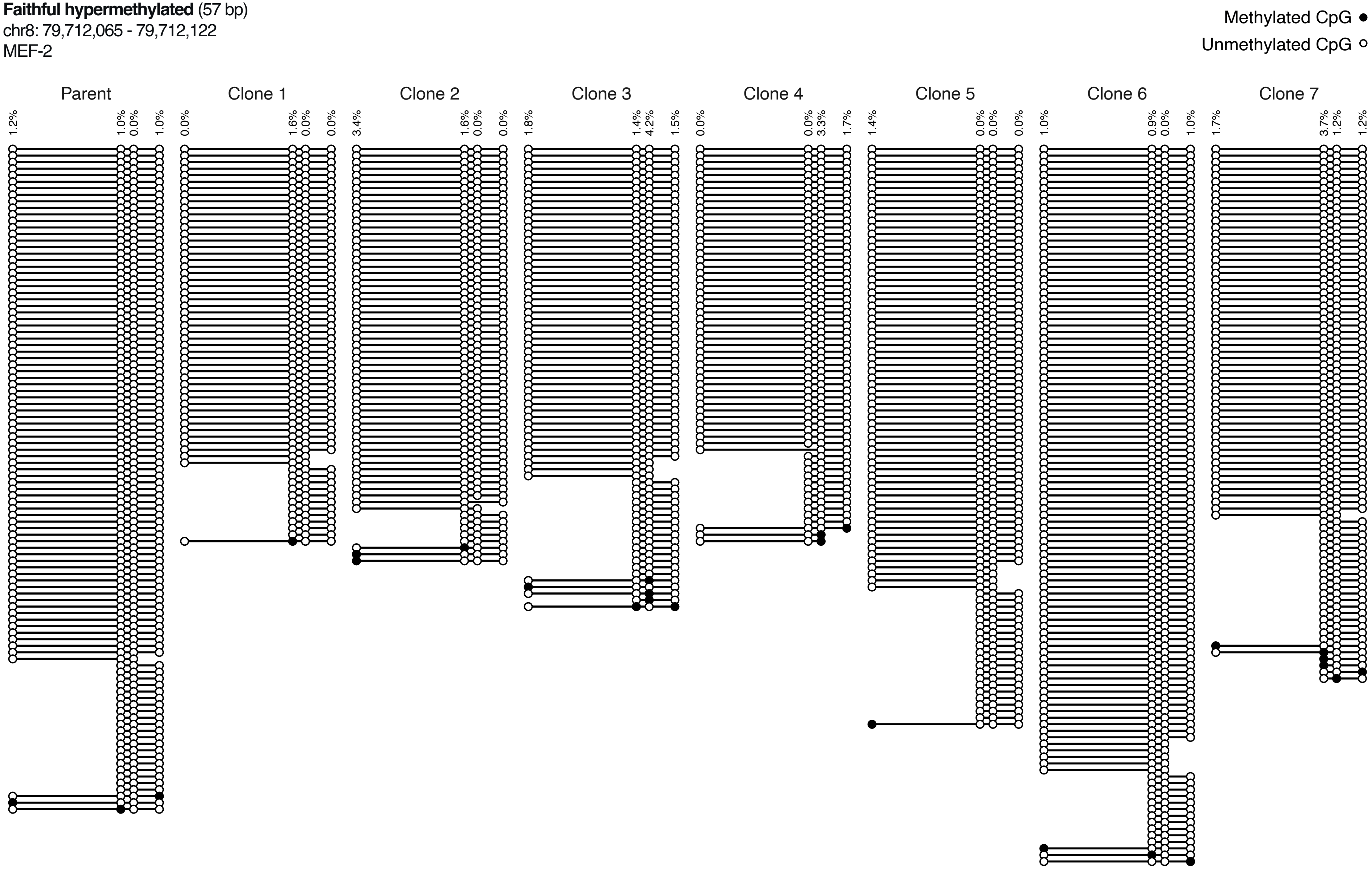

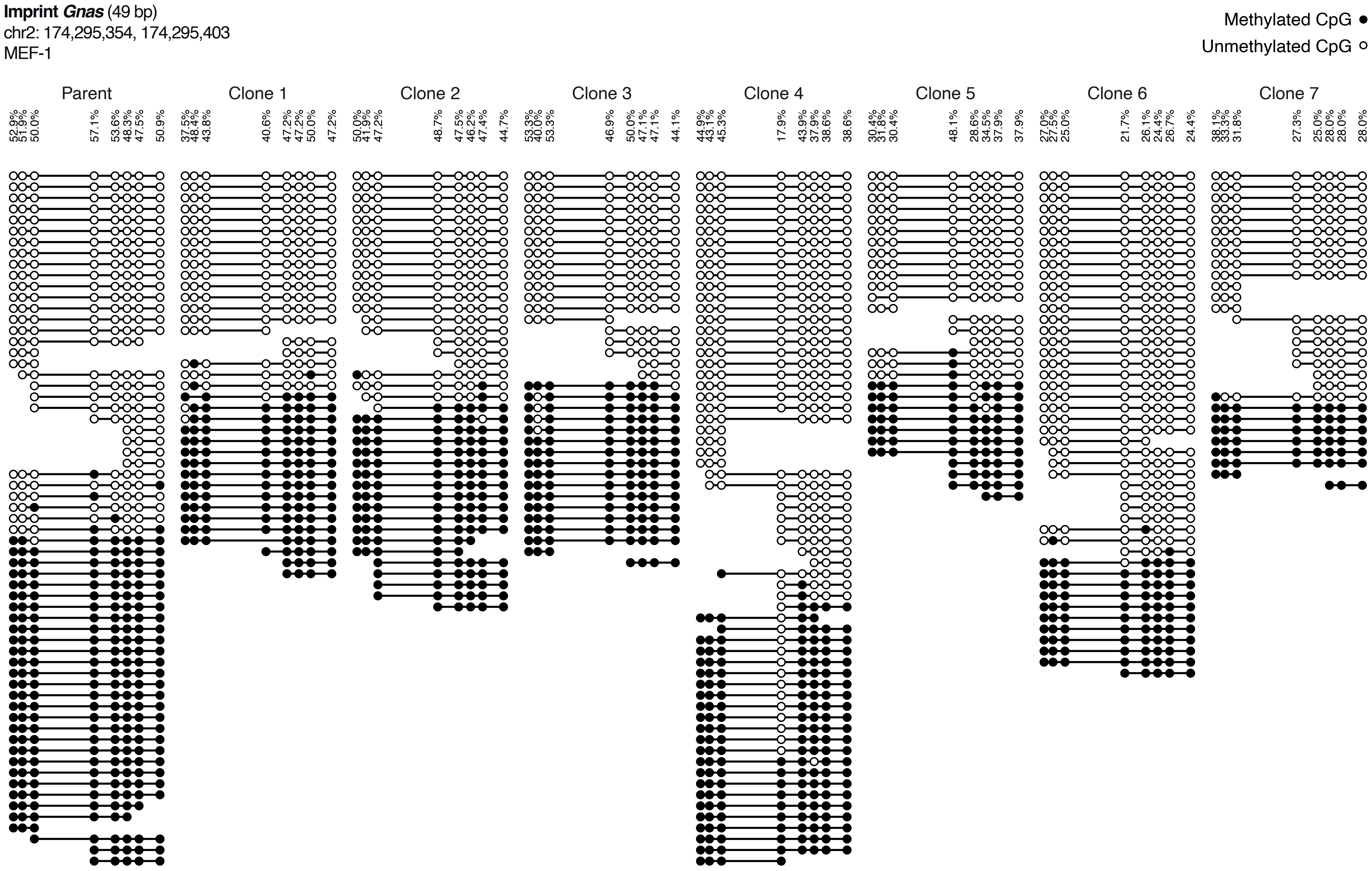

Imprint *Gnas* (49 bp)
chr2: 174,295,354, 174,295,403
MEF-1
Methylated CpG
Unmethylated CpG
Parent
Clone 1
Clone 2
Clone 3
Clone 4
Clone 5
Clone 6
Clone 7

Imprint *Kcnq1* (70 bp)
chr7: 143,295,570 - 143,295,640
MEF-1

Methylated CpG ●
Unmethylated CpG ○

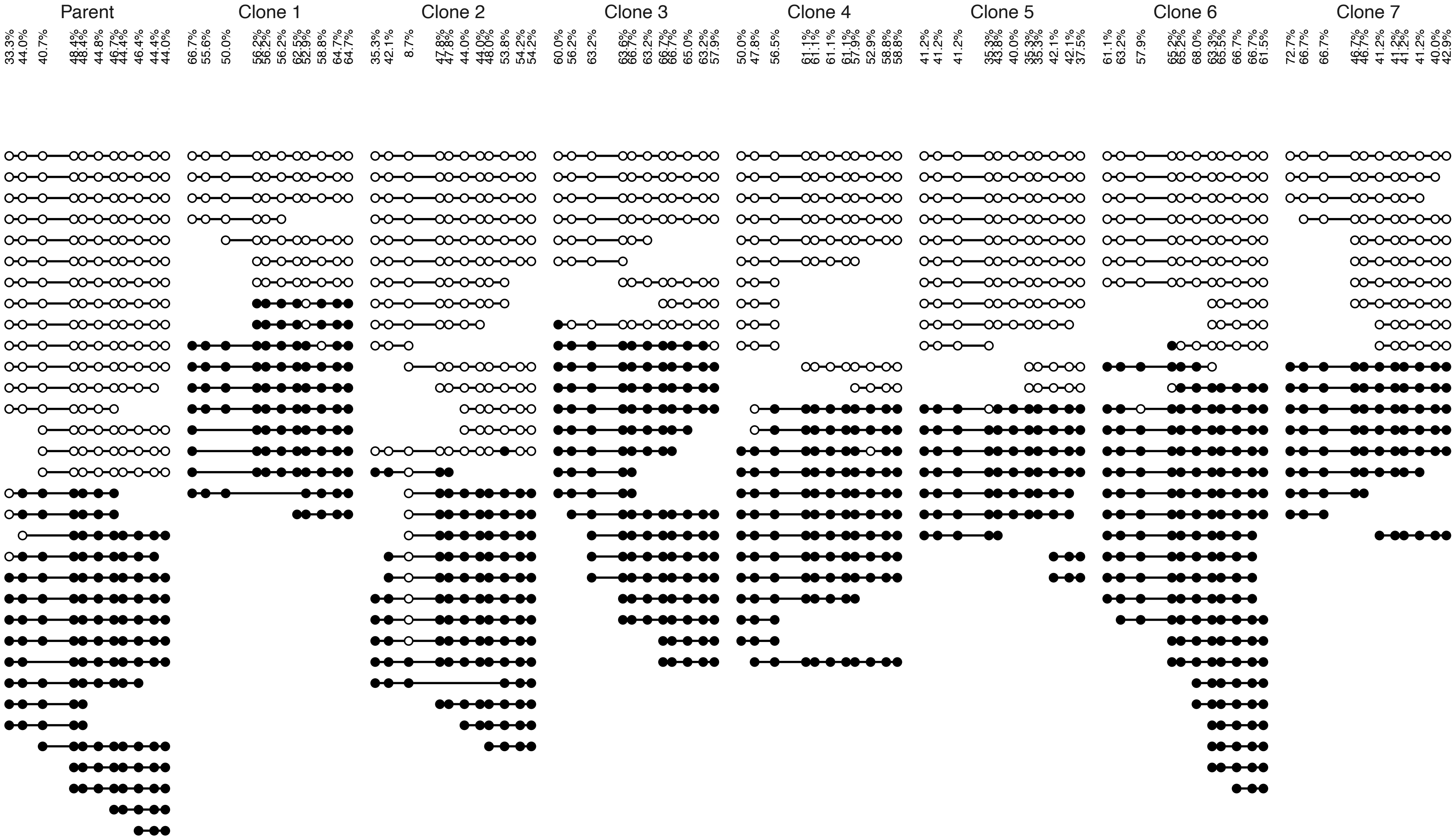

Imprint *Gnas* (49 bp)
chr2: 174,295,354, 174,295,403
MEF-2

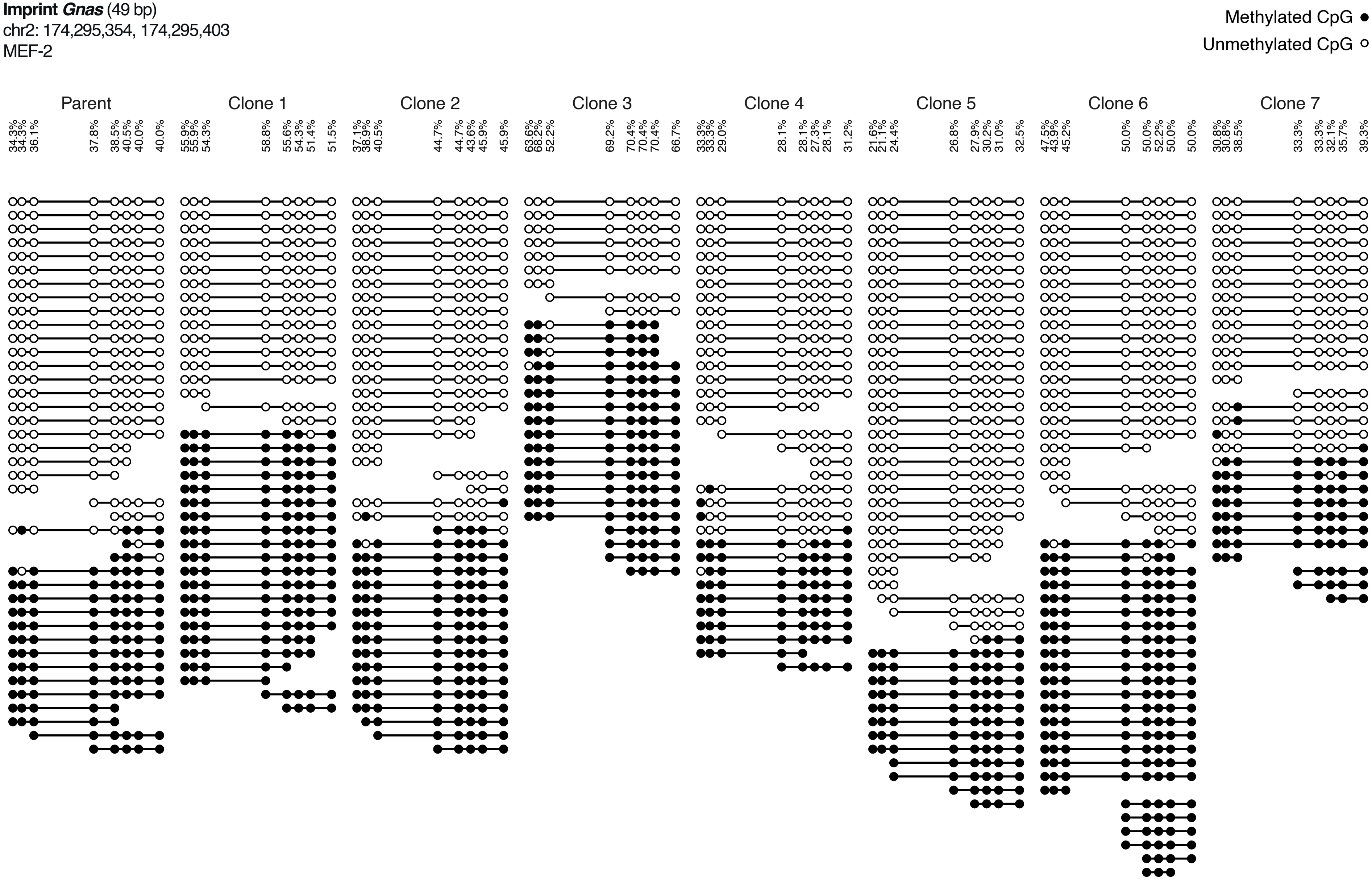

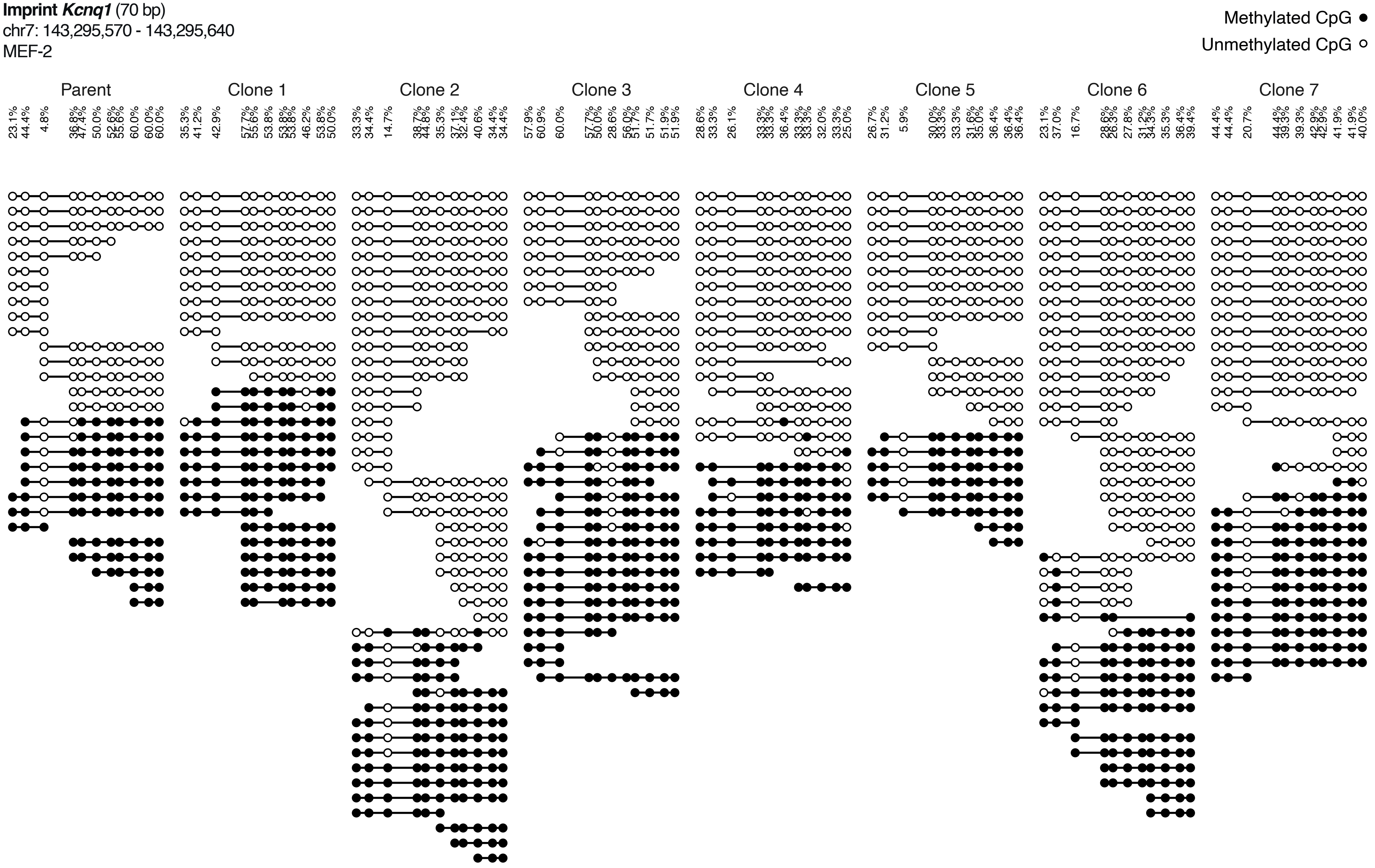
Imprint *Kcnq1* (70 bp)
chr7: 143,295,570 - 143,295,640
MEF-2
Methylated CpG ●
Unmethylated CpG ○
Parent
23.1% 44.4% 4.8% 36.8% 47.4% 50.0% 52.6% 55.6% 60.0% 60.0% 60.0%
Clone 1
35.3% 41.2% 42.9% 57.7% 55.6% 53.8% 53.8% 53.8% 46.2% 53.8% 50.0%
Clone 2
33.3% 34.4% 14.7% 38.7% 44.8% 35.3% 37.1% 32.4% 40.6% 34.4% 34.4%
Clone 3
57.9% 60.9% 60.0% 57.7% 50.0% 28.6% 56.0% 51.7% 51.7% 51.9% 51.9%
Clone 4
28.6% 33.3% 26.1% 33.3% 33.3% 36.4% 33.3% 33.3% 32.0% 33.3% 25.0%
Clone 5
26.7% 31.2% 5.9% 30.0% 33.3% 33.3% 31.6% 35.0% 36.4% 36.4% 36.4%
Clone 6
23.1% 37.0% 16.7% 28.6% 26.3% 27.8% 31.2% 34.3% 35.3% 36.4% 39.4%
Clone 7
44.4% 44.4% 20.7% 44.4% 39.3% 39.3% 42.9% 42.9% 41.9% 41.9% 40.0%

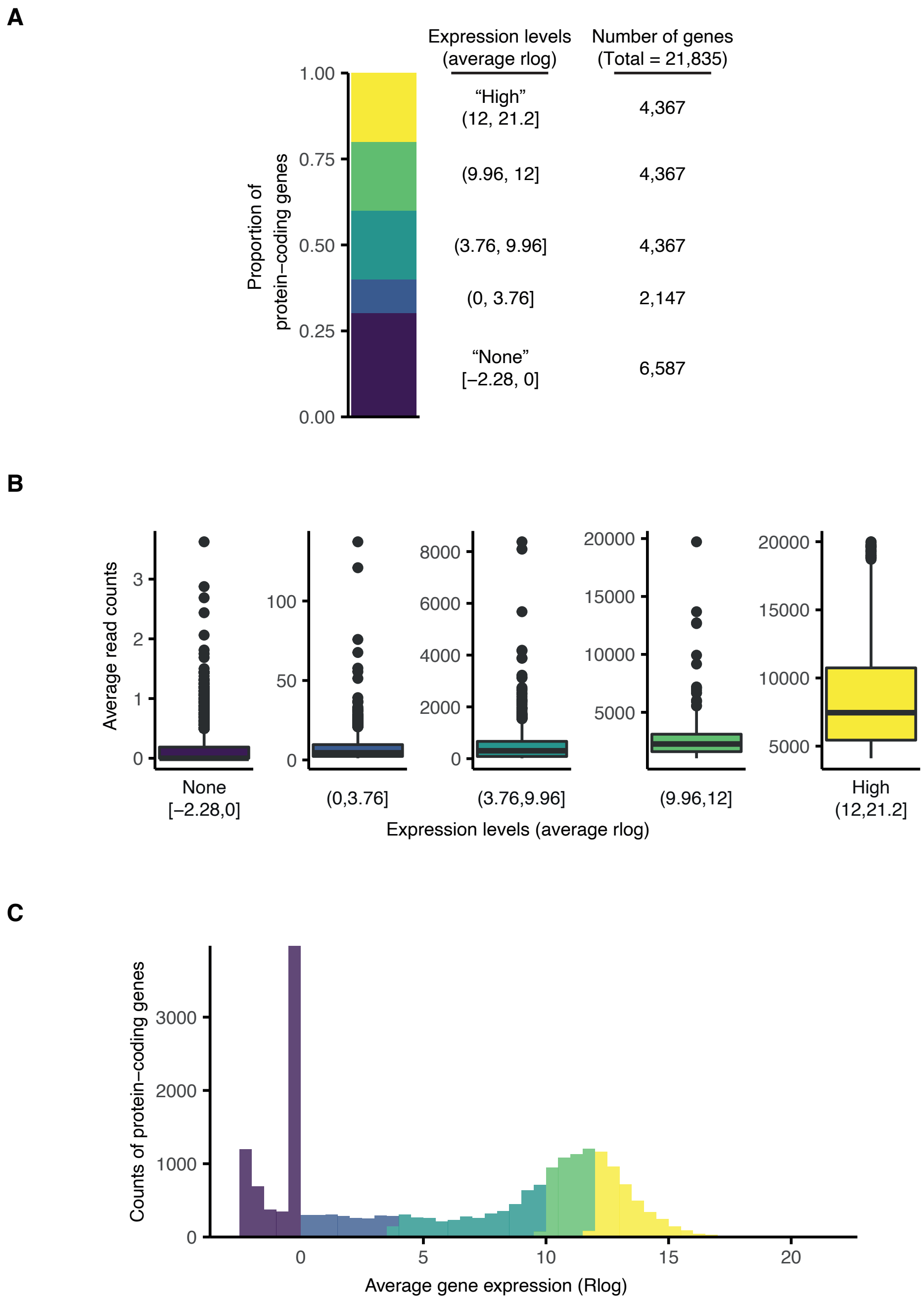

Supplementary Fig. 20: Classifying expression levels in MEFs. (A) Bar plot showing proportions of protein-coding gene expression levels as determined by average rlog expression ranges quantified using all MEF-1 and MEF-2 RNA-seq datasets. The annotation of all 21,835 protein-coding genes in the mouse genome is derived from Ensembl release 102. (B) Boxplots showing average read counts of the different expression levels. (C) Distribution of average gene expression rlog values coloured by gene expression levels. For (A) and (B), square brackets indicate that the boundary data point is included within the indicated dataset (inclusive), while the parentheses indicate that the boundary data point is not included (exclusive). The number of genes per expression level category is reported in (A) and is the same for both (B) and (C). For (B), the box of the boxplot shows the 25th, 50th, and 75th percentiles; the whiskers extend to 1.5 * IQR beyond the edges of the box (where IQR = 75th −25th percentile), with outliers shown as dots.

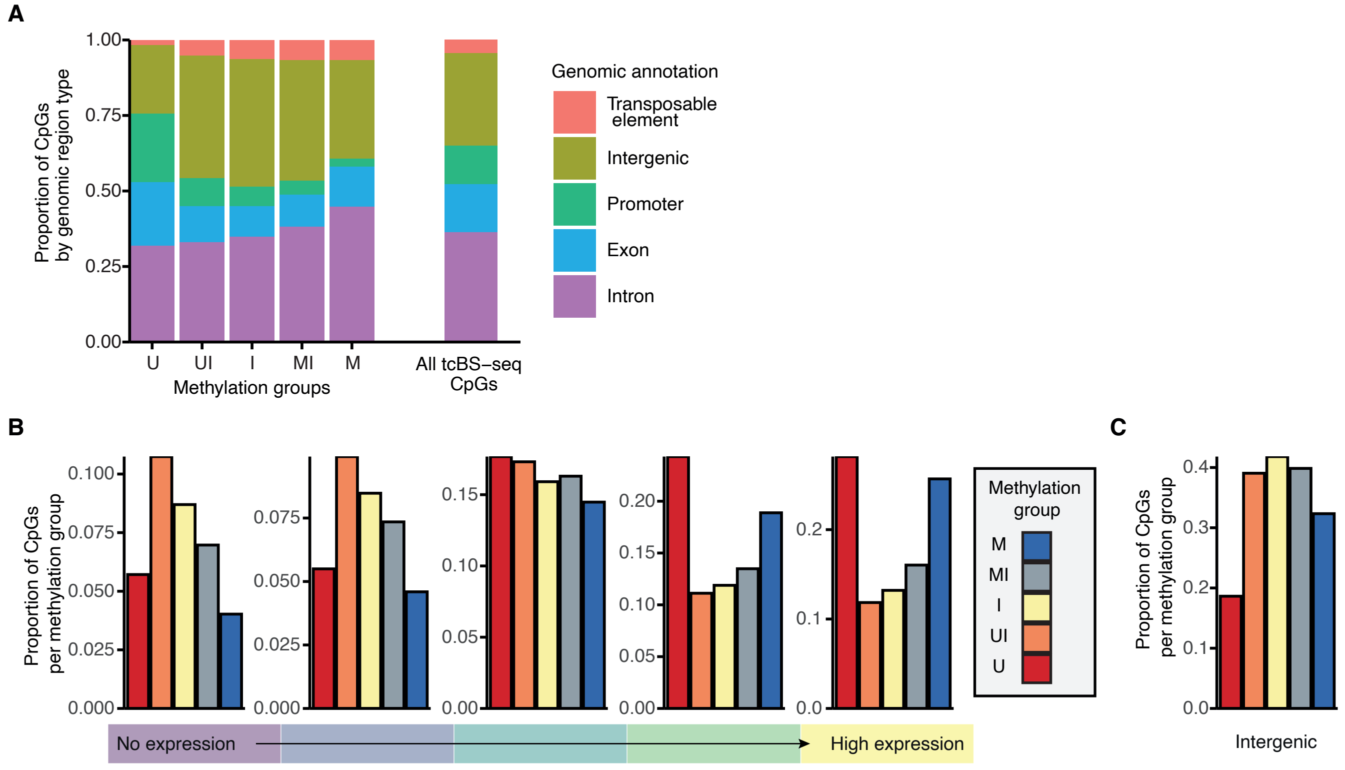

Supplementary Fig. 21: CpGs that exhibit intermediate methylation associate with transcriptional inactivity in MEFs. (A) Relative distribution of different genomic annotations amongst the methylation groups and all CpGs assessed. (B) Proportion of CpGs in methylation groups that overlap with protein-coding genes of varying expression levels. For this overlap, 1000bp before the transcription start site is included at all protein-coding genes to account for CpGs that overlap with promoters. (C) Proportion of CpGs in methylation groups that are intergenic. CpGs that do not overlap with a protein-coding gene or promoter are classified as intergenic. Gene expression levels are represented by colours ranging from purple (no expression) to yellow (high expression).

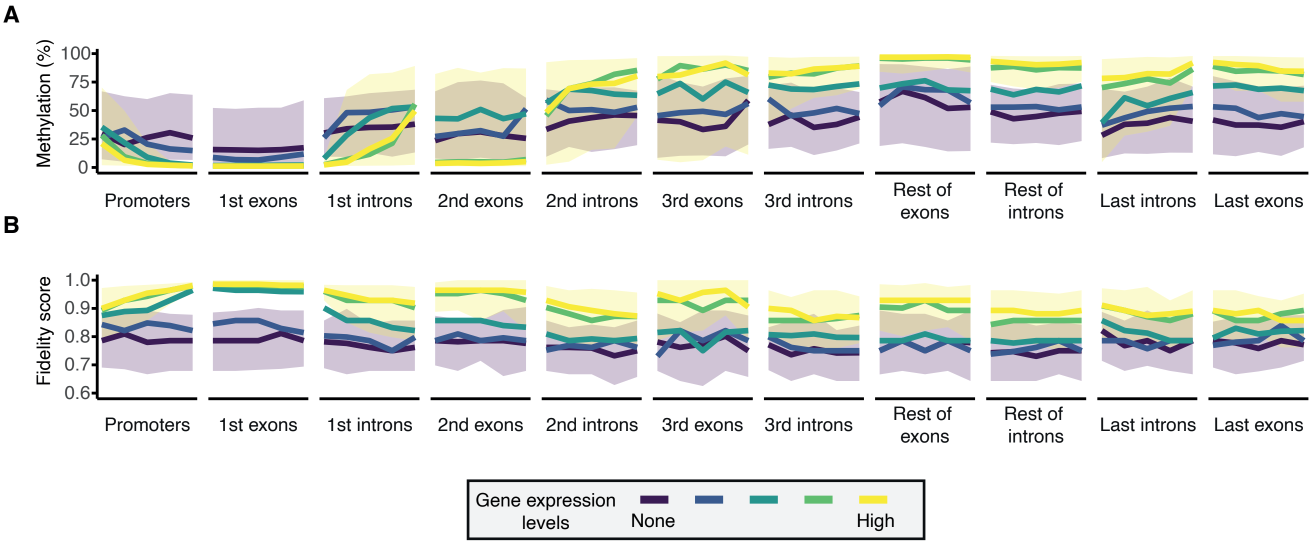

Supplementary Fig. 22: Methylation levels and methylation fidelity at protein-coding genes of varying expression in MEFs. (A) Methylation levels and (B) fidelity score characterised along regions of protein-coding genes. Each genic region is split into five tiles at which median (lines) or interquartile range (ribbons) of methylation or fidelity score is shown. Only genic regions covered by at least 3 CpGs are considered; single-exon genes are excluded. Gene expression levels are represented by colours ranging from purple (no expression) to yellow (high expression). Interquartile range ribbons for both methylation and fidelity score are only shown for the genes that are not expressed (light purple) or highly expressed (light yellow).

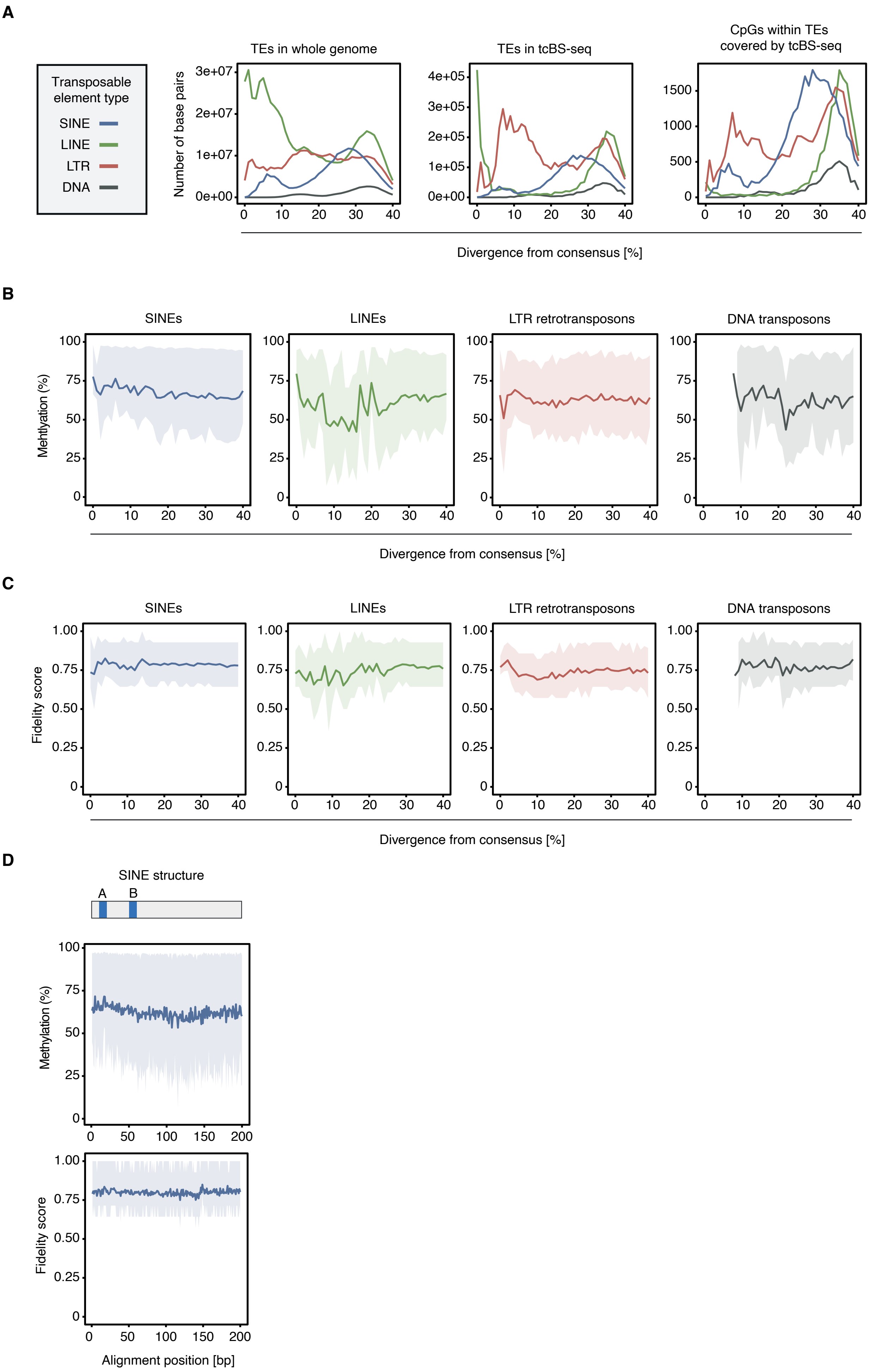
A
Transposable element type
SINE
LINE
LTR
DNA
TEs in whole genome
TEs in tcBS-seq
CpGs within TEs covered by tcBS-seq
Number of base pairs
3e+07
2e+07
1e+07
0e+00
4e+05
3e+05
2e+05
1e+05
0e+00
1500
1000
500
0
0
10
20
30
40
Divergence from consensus [%]
B
SINEs
LINEs
LTR retrotransposons
DNA transposons
Methlyation (%)
100
75
50
25
0
Divergence from consensus [%]
C
SINEs
LINEs
LTR retrotransposons
DNA transposons
Fidelity score
1.00
0.75
0.50
0.25
0
Divergence from consensus [%]
D
SINE structure
A
B
Methylation (%)
Fidelity score
0
50
100
150
200
Alignment position [bp]

Supplementary Fig. 23: Methylation fidelity at transposable elements. (A) Plots showing base pair counts for TEs in the whole genome (left), TEs represented by the tcBS-seq data (middle), and the CpGs within TEs represented by the tcBS-seq data, with varying percent divergences from the consensus sequence. TE types are represented by colour: SINEs in blue; LINEs in green; LTR retrotransposons (LTR) in red; DNA transposons (DNA) in grey. (B and C) Plots of methylation level (B) or fidelity score (C) versus percent divergence from the consensus sequence for different TE types. (D) Plots of methylation level and fidelity score versus the position of a CpG within a SINE. Top bar shows the archetypal SINE structure relative to the alignment position on the x-axis of the plots below, with A- and B-block promoter regions coloured in blue. For all plots, methylation levels and fidelity score are calculated as averages across CpGs in each TE; lines represent mean values, while ribbons show the interquartile range.

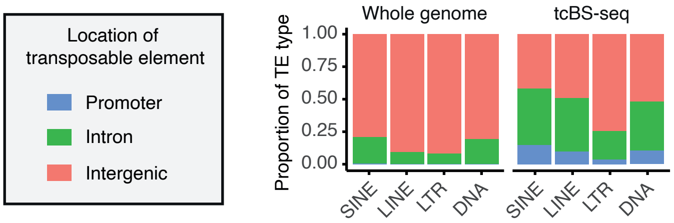

Supplementary Fig. 24: Enrichment of genically located transposable elements in tcBS-seq. Proportion of TE types across three different genomic locations (intergenic, intron, and promoter) both genome-wide (left) and in the tcBS-seq data (right). Genomic locations are represented by colour: promoter in blue; intron in green; intergenic in orange.

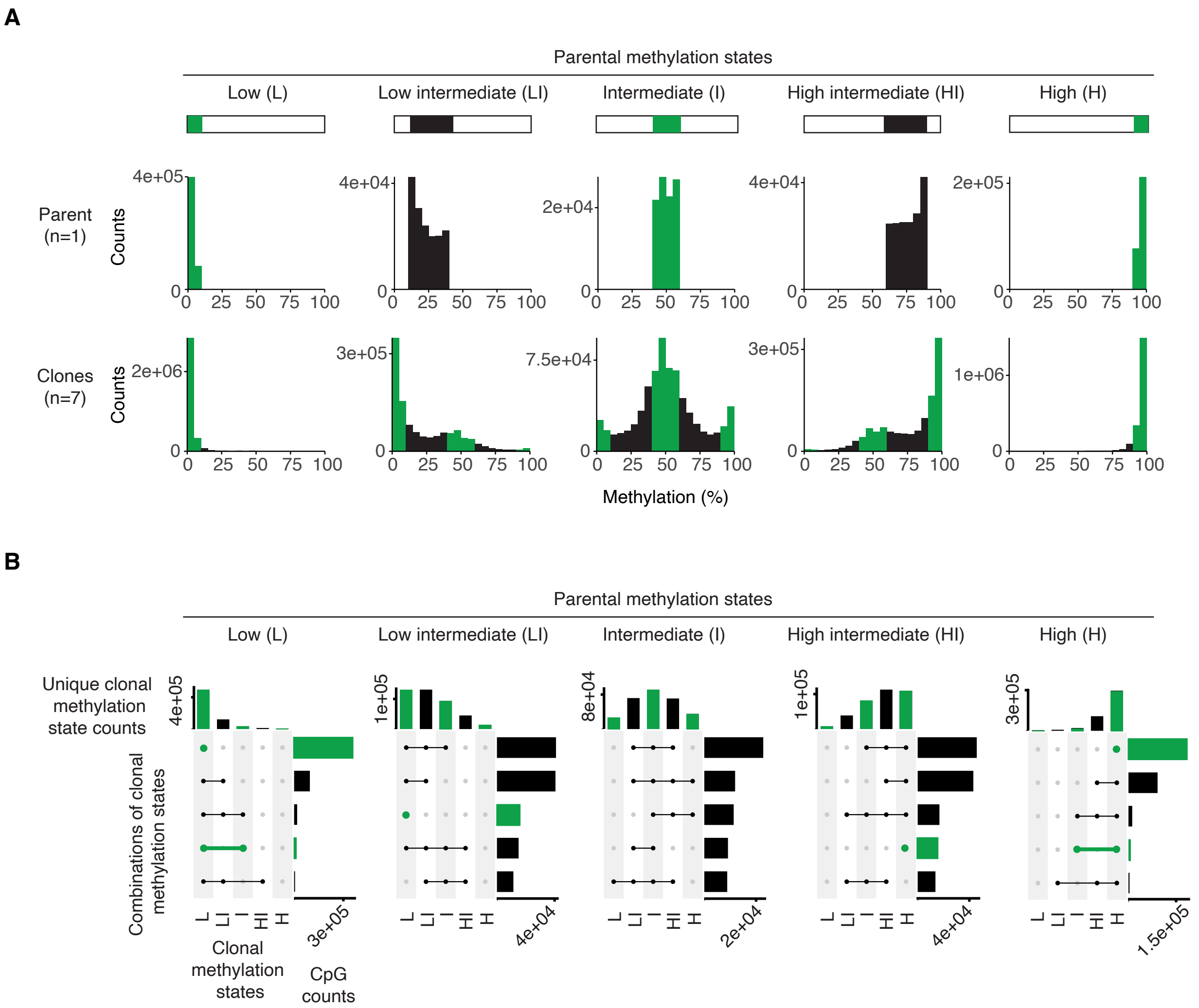

Supplementary Fig. 25: Intermediately methylated CpGs are prone to probabilistic inheritance through clonal expansion in MEF-2 cell lines. (A) Clonal line methylation distributions from different parental line methylation states for the MEF-2 cell lines. (B) UpSet plots of clonal line methylation states per CpG from different parental line methylation states for the MEF-2 cell lines. In each panel, the top bar plots show the number of unique clonal methylation states represented per CpG. The horizontal bar plots show the CpG counts that exhibit a certain combination of clonal methylation states. Only the five most representative clonal methylation state combinations are shown. Green bars represent cases of potential faithful methylation inheritance because this kind of methylation inheritance will only result in 0%, 50%, or 100% methylation states in the clonal lines. Similarly, green dots and lines in the UpSet plots represent cases and combinations of potential faithful methylation inheritance. Methylation states are defined quantitatively as the following: Low = 0-10%, Low intermediate = 10-40%, Intermediate = 40-60%, High intermediate = 60-90%, High = 90-100% methylation.

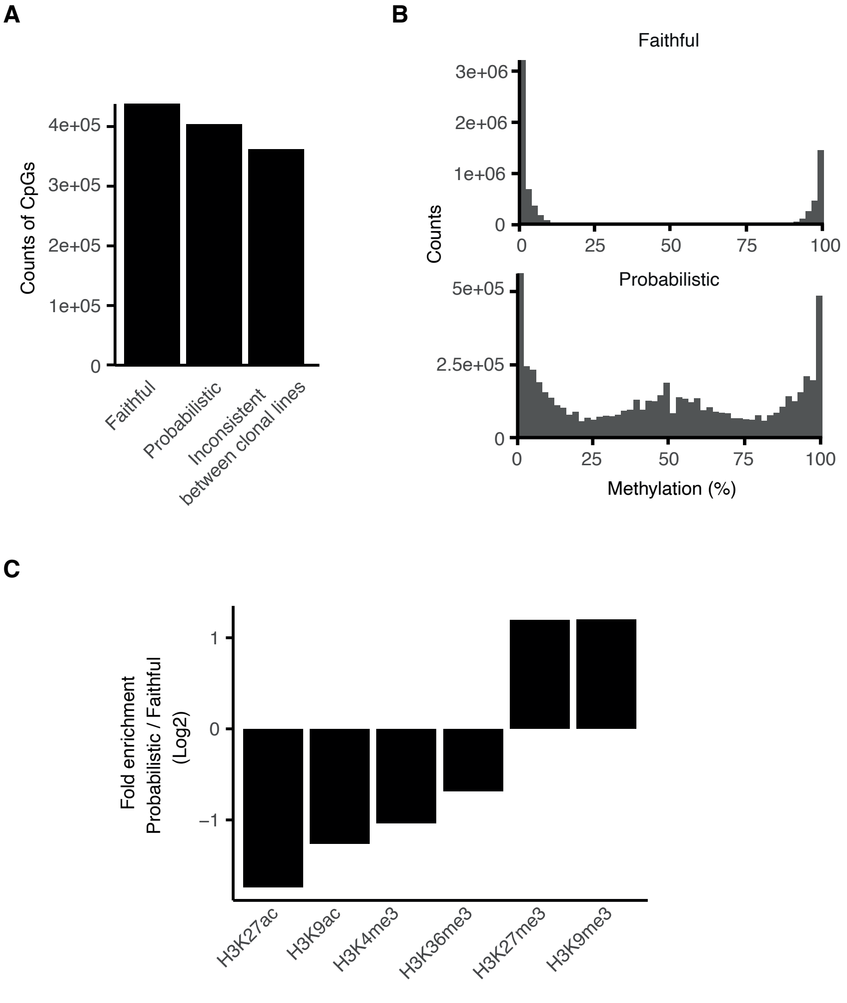

Supplementary Fig. 26: Probabilistically methylated CpGs associate with repressive histone tail modifications H3K27me3 and H3K9me3. A) Counts of faithfully and probabilistically methylated CpGs. CpGs are defined as faithful if all clonal lines exhibit [0-10], (40-60], or (90-100] % methylation. If at least one clonal line exhibits (10-40] or (60-90] % methylation in both MEF-1 and MEF-2, we define the CpGs as probabilistically methylated. CpGs that are inconsistently characterised as faithful or probabilistic between MEF-1 and MEF-2 are not considered for further analyses. (B) Methylation distributions of faithful and probabilistic CpGs across all MEF-1 and MEF-2 cell lines. (C) Enrichment of histone tail modification peak overlap with probabilistic versus faithful CpGs.

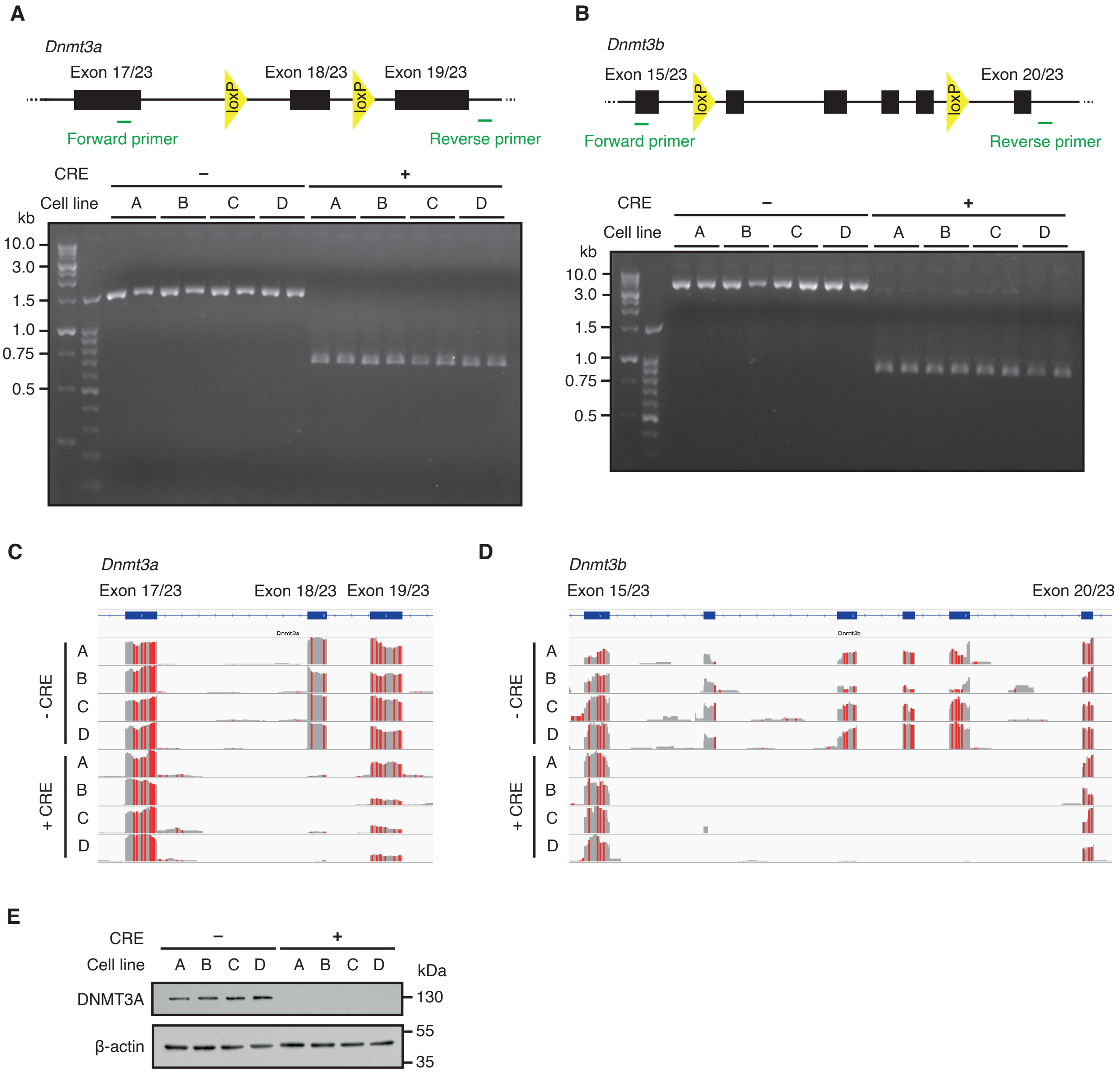

Supplementary Fig. 27: Confirmation of induced Dnmt3a/3b DKO in primary MEFs. (A) Top: diagram showing the location of the loxP sites flanking exon 18 of *Dnmt3a*, as well as the location of the primers used to confirm the knockout (KO). Bottom: agarose gel image of a PCR to confirm the TAT-CRE induced KO of *Dnmt3a* in the DNA. (B) Top: diagram showing the location of the loxP sites flanking exons 16-19 of *Dnmt3b*, as well as the location of the primers used to confirm the KO. Bottom: agarose gel image of a PCR to confirm the TAT-CRE induced KO of *Dnmt3b* in the DNA. (C and D) IGV screenshots of RNA-seq data showing the absence of transcripts that include the loxP flanked exons for both *Dnmt3a* and *Dnmt3b*. (E) Western blots of untreated and TAT-CRE-treated cell lines for DNMT3A and loading control ß-actin. PCR gel images from (A) and (B) were each performed with four biological replicates (labelled as A, B, C, and D) and two technical replicates; the western blot from (E) was performed with four biological replicates (labelled as A, B, C, and D).

Supplementary Figure 28
Uncropped western blots corresponding to Supplementary Fig. 27E.

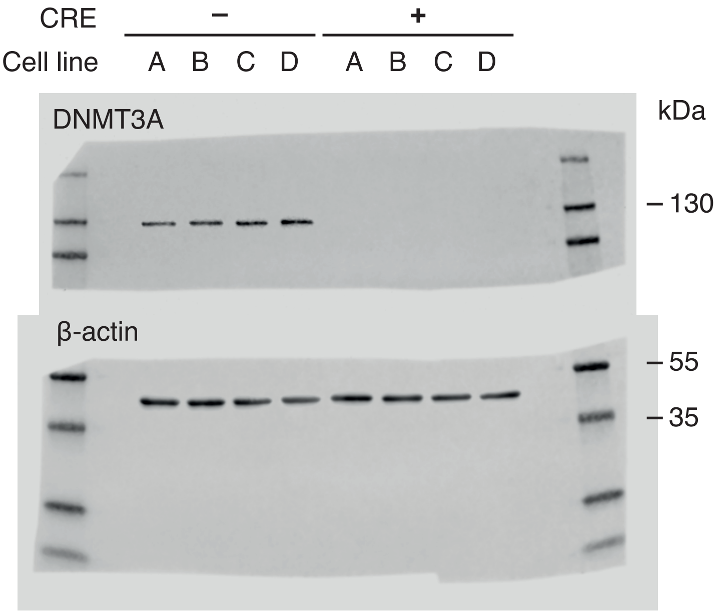

Supplementary Table 1: Target capture bisulfite sequencing (tcBS-seq) coverage by CpG.

Region Type	Annotation / Dataset	Region	Number of CpGs in the genome (Total = 21,353,092)	Percentage of CpGs in the genome (Column 4 / 21,353,092)	Number of CpGs represented by tcBS-seq (Total = 1,203,687)	Percentage of CpGs represented by tcBS-seq (Column 6 / 1,203,687)	Percentage of CpGs represented by tcBS-seq by region (Column 6 / Column 4)
Genic	GENCODE (M20)	Promoters (1kb prior to TSS)	1,536,326	7.2%	320,221	26.6%	20.84%
		Exons	2,396,981	11.2%	334,342	27.8%	13.95%
		Introns	10,005,524	46.9%	621,419	51.6%	6.21%
Transposable element	RepeatMasker	SINEs	1,908,515	8.9%	23,982	2.0%	1.26%
		LINEs	3,153,540	14.8%	6,268	0.5%	0.20%
		LTRs	2,606,533	12.2%	22,043	1.8%	0.85%
		DNA transposons	205,354	1.0%	3,367	0.3%	1.64%
CpG island	UCSC	CpG islands	1,076,260	5.0%	280,479	23.3%	26.06%
Replication timing	Repliseq (MEFs) GSE196749	Early fraction	11,990,691	56.2%	956,368	79.5%	7.98%
		Late fraction	8,407,912	39.4%	203,226	16.9%	2.42%

Supplementary Table 2: Target capture bisulfite sequencing (tcBS-seq) coverage by region.

Region Type	Annotation	Region	Total number of regions with at least one CpG in the mm10 genome	Number of regions with tcBS-seq methylation data	Regions with tcBS-seq methylation data (%)
Genic	GENCODE (M20)	Promoters (1kb prior to TSS)	74,392	20,273	27.3%
		Exons	240,736	33,321	13.8%
		Introns	173,791	39,879	22.9%
Transposable element	RepeatMasker	SINEs	1,008,782	19,287	1.9%
		LINEs	617,830	7,098	1.1%
		LTRs	618,058	13,443	2.2%
		DNA transposons	112,711	2,851	2.5%
CpG island	UCSC	CpG islands	15,997	11,360	71.0%

Supplementary Table 3: Publicly available bisulfite-sequencing and RNA-sequencing datasets.

Cell type	Library Prep Type	GEO / ENCODE accession	ENCODE (Bed file)
Primary MEFs	WGBS	GSM2648457	-
3T3 immortalised MEFs	WGBS	GSM4942823, GSM4942824, GSM4942825	-
E13.5 mouse limb	WGBS	ENCSR700FCF	ENCFF556ENA, ENCFF834PXN
E14.5 mouse limb	WGBS	ENCSR099RYD	ENCFF176VLZ, ENCFF038IVS

Supplementary Data Table 4: Number of genes and genic regions represented in methylation data.

Genic region	Cell type	Promoter	1st exon	1st intron	2nd exon	2nd intron	3rd exon	3rd intron	Rest of exons (number of exons)	Rest of introns (number of introns)	Last intron	Last exon
**Genes represented in methylation data (#)**	MEF	9,583	6,327	8,797	2,194	3,227	1,109	1,980	2,391 (3,421)	4,511 (8,677)	2,395	2,668

Supplementary Table 5: Counts and ratios of faithful and probabilistic CpG overlap with various histone tail modification peaks.

**Histone tail modification**	**Faithful**<br>**Total # CpGs= 437,834**<br># overlapped CpGs<br>(% overlapped CpGs)	**Probabilistic**<br>**Total # CpGs = 403,672**<br># overlapped CpGs<br>(% overlapped CpGs)	**ENCODE (Bed file) E13.5 Limb**	**ENCODE (Accession) E13.5 Limb**
H3K27ac	167,886<br>(38.3%)	46,448<br>(11.5%)	ENCFF283BKX	ENCSR905FFU
H3K9ac	230,562<br>(52.7%)	88,587<br>(21.9%)	ENCFF733UCO	ENCSR462BZP
H3K4me3	266,086<br>(60.8%)	119,898<br>(29.7%)	ENCFF829LEB	ENCSR416OYH
H3K36me3	36,409<br>(8.3%)	20,871<br>(5.2%)	ENCFF404DJU	ENCSR639IQR
H3K27me3	37,070<br>(8.5%)	78,360<br>(19.4%)	ENCFF223KSJ	ENCSR709CLU
H3K9me3	3,019<br>(0.7%)	6,407<br>(1.6%)	ENCFF293BQI	ENCSR022DED
