# Supplementary material for: Epigenetic inheritance is unfaithful at intermediately methylated CpG sites
Source: Nat Commun. 2023 Sep 2;14:5336. doi: 10.1038/s41467-023-40845-2 (PMC10475082; doi:10.1038/s41467-023-40845-2)
Supplement: Supplementary file 3 — Reporting Summary [file 41467_2023_40845_MOESM3_ESM.pdf]

nature portfolio

Corresponding author(s): Anne C Ferguson-Smith, Felipe Karam Teixeira

Last updated by author(s): Feb 6, 2023

Reporting Summary

Nature Portfolio wishes to improve the reproducibility of the work that we publish. This form provides structure for consistency and transparency in reporting. For further information on Nature Portfolio policies, see our Editorial Policies and the Editorial Policy Checklist.

Statistics

For all statistical analyses, confirm that the following items are present in the figure legend, table legend, main text, or Methods section.

n/a	Confirmed	
☐	☒	The exact sample size (*n*) for each experimental group/condition, given as a discrete number and unit of measurement
☐	☒	A statement on whether measurements were taken from distinct samples or whether the same sample was measured repeatedly
☐	☒	The statistical test(s) used AND whether they are one- or two-sided<br>*Only common tests should be described solely by name; describe more complex techniques in the Methods section.*
☒	☐	A description of all covariates tested
☒	☐	A description of any assumptions or corrections, such as tests of normality and adjustment for multiple comparisons
☐	☒	A full description of the statistical parameters including central tendency (e.g. means) or other basic estimates (e.g. regression coefficient) AND variation (e.g. standard deviation) or associated estimates of uncertainty (e.g. confidence intervals)
☐	☒	For null hypothesis testing, the test statistic (e.g. *F*, *t*, *r*) with confidence intervals, effect sizes, degrees of freedom and *P* value noted<br>*Give P values as exact values whenever suitable.*
☒	☐	For Bayesian analysis, information on the choice of priors and Markov chain Monte Carlo settings
☐	☒	For hierarchical and complex designs, identification of the appropriate level for tests and full reporting of outcomes
☒	☐	Estimates of effect sizes (e.g. Cohen's *d*, Pearson's *r*), indicating how they were calculated

Our web collection on statistics for biologists contains articles on many of the points above.

Software and code

Policy information about availability of computer code

Data collection	No software was used for data collection.

Data analysis	All code used to perform the analyses is available on GitHub (https://github.com/AFS-lab/methylation_fidelity).<br>Trim Galore (v0.6.0)<br>Bismark (v0.22.3)<br>R (v3.6.1)<br>Salmon (v1.5.2)<br>DESeq2 (v1.24.0)<br>SeqPlots (v3.0.12)<br>ggplot2 (v3.3.5)<br>readr (v2.0.1)<br>dplyr (v1.0.7)<br>Python (v3.5.3)<br>GNU bash (v4.4.12)<br>GNU Awk (v4.1.4)<br>GenomicRanges (v1.36.1)<br>UpSetR (v1.4.0)<br>RColorBrewer (v1.1-2)

For manuscripts utilizing custom algorithms or software that are central to the research but not yet described in published literature, software must be made available to editors and reviewers. We strongly encourage code deposition in a community repository (e.g. GitHub). See the Nature Portfolio guidelines for submitting code & software for further information.

Data

Policy information about availability of data

All manuscripts must include a data availability statement. This statement should provide the following information, where applicable:

- Accession codes, unique identifiers, or web links for publicly available datasets
- A description of any restrictions on data availability
- For clinical datasets or third party data, please ensure that the statement adheres to our policy

All raw and processed sequencing data generated in this study have been submitted to the NCBI Gene Expression Omnibus (GEO; https://www.ncbi.nlm.nih.gov/geo/) under accession number GSE234695 (parental/clonal tcBS-seq and total RNA-seq; Dnmt3a/3b DKO tcBS-seq) and to the Sequence Read Archive (SRA, https://www.ncbi.nlm.nih.gov/sra) under accession number PRJNA980423 (Dnmt3a/3b DKO RNA-seq).

Human research participants

Policy information about studies involving human research participants and Sex and Gender in Research.

Reporting on sex and gender	Not applicable
Population characteristics	Not applicable
Recruitment	Not applicable
Ethics oversight	Not applicable

Note that full information on the approval of the study protocol must also be provided in the manuscript.

Field-specific reporting

Please select the one below that is the best fit for your research. If you are not sure, read the appropriate sections before making your selection.

☒ Life sciences ☐ Behavioural & social sciences ☐ Ecological, evolutionary & environmental sciences

For a reference copy of the document with all sections, see nature.com/documents/nr-reporting-summary-flat.pdf

Life sciences study design

All studies must disclose on these points even when the disclosure is negative.

Sample size	No statistical methods were used to predetermine sample size. Information about sample collection and replication is presented in the Methods section. We performed methylation and RNA sequencing experiments on 14 clones derived from 2 parental cell lines with the intention to maximise the number of experiments while keeping costs reasonable. Additionally, we performed methylation and RNA sequencing experiments on 4 biological replicates of double knockout and corresponding control cell lines.
Data exclusions	Methylation data with fewer than 10 reads per CpG site were excluded because they may not accurately reflect methylation statuses. Methylation data at aneuploidic chromosomes were excluded because they do not fall within the framework of the study.

Replication	We performed methylation and RNA sequencing experiments on 14 clones derived from 2 parental cell lines, as well as 4 biological replicates of double knockout and corresponding control cell lines.
Randomization	Randomization was not used since the methods described in this study are intended for exploratory and unsupervised analyses.
Blinding	Blinding was not used since the methods described in this study are intended for exploratory and unsupervised analyses.

Reporting for specific materials, systems and methods

We require information from authors about some types of materials, experimental systems and methods used in many studies. Here, indicate whether each material, system or method listed is relevant to your study. If you are not sure if a list item applies to your research, read the appropriate section before selecting a response.

Materials & experimental systems

n/a	Involved in the study	
☐	☒	Antibodies
☐	☒	Eukaryotic cell lines
☒	☐	Palaeontology and archaeology
☐	☒	Animals and other organisms
☒	☐	Clinical data
☒	☐	Dual use research of concern

Methods

n/a	Involved in the study	
☒	☐	ChIP-seq
☒	☐	Flow cytometry
☒	☐	MRI-based neuroimaging

Antibodies

Antibodies used	anti-DNMT3a (abcam, ab188470), anti-b-actin (abcam, ab8227), rabbit IgG-HRP (Agilent, cat no. P044801-2)
Validation	No validation was performed.

Eukaryotic cell lines

Policy information about cell lines and Sex and Gender in Research

Cell line source(s)	MEF-1 (male) and MEF-2 (female) primary cell lines were established from sibling E13.5 C57BL/6J mouse embryos. These primary MEF-1 and MEF-2 cell lines were immortalised by serial passaging the cells through crisis phase to establish the "parental" MEF-1 and MEF-2 cell lines from which subclones were derived by randomly selecting single cells by flow cytometry.<br>Primary MEF cell lines A (male), B (female), C (male), and D (female) were established from Dnmt3a(flox/flox)3b(flox/flox) E13.5 mouse embryos.
Authentication	The cell lines have not been authenticated
Mycoplasma contamination	All cell lines tested negative for mycoplasma contamination
Commonly misidentified lines (See ICLAC register)	No commonly misidentified lines were used in this study

Animals and other research organisms

Policy information about studies involving animals; ARRIVE guidelines recommended for reporting animal research, and Sex and Gender in Research

Laboratory animals	Mouse work was conducted under project licenses from the UK government Home Office (project license numbers: PC9886123, PC213320E, and PP8193772). Mice were housed in a temperature and humidity-controlled room under 12 hr light / 12 hr dark cycles and fed a standard chow diet ad libitum. Post-implantation embryos (E13.5 for mouse embryonic fibroblasts) and blastocysts (E3.5 for mouse embryonic stem cells) were collected by natural mating, and the plugged date of conception was considered E0.5. Dnmt3aflox/flox3bflox/flox mice were obtained from RIKEN BioResource Research Center (BRC) and maintained on a C57BL/6 mouse background.
Wild animals	Not applicable.
Reporting on sex	We used the genomic enrichment across the X chromosome, as well as the following primers to amplify the SRY gene on the Y chromosome to determine the difference in sex between the parental MEF lines:<br>SRY_F: GCAGGCTGTAAAATGCCACT

	SRY_R: TTCCAGGAGGCACAGAGATT<br>MEF-1 is male and MEF-2 is female. We did not have any preconceived consideration of sex in this study and removed the sex chromosomes from the analysis.
Field-collected samples	Not applicable.
Ethics oversight	Mouse work was conducted under project licenses from the UK government Home Office (project license numbers: PC9886123, PC213320E, and PP8193772).

Note that full information on the approval of the study protocol must also be provided in the manuscript.
